# Subtle Longitudinal Alterations in Env Sequence Potentiate Differences in Sensitivity to Broadly Neutralizing Antibodies following Acute HIV-1 Subtype C Infection

**DOI:** 10.1128/jvi.01270-22

**Published:** 2022-12-01

**Authors:** Tawanda Mandizvo, Nombali Gumede, Bongiwe Ndlovu, Siphiwe Ndlovu, Jaclyn K. Mann, Denis R. Chopera, Lanish Singh, Krista L. Dong, Bruce D. Walker, Zaza M. Ndhlovu, Christy L. Lavine, Michael S. Seaman, Kamini Gounder, Thumbi Ndung’u

**Affiliations:** a Africa Health Research Institute, Durban, South Africa; b HIV Pathogenesis Programme, Doris Duke Medical Research Institute, University of KwaZulu-Natal, Durban, South Africa; c Ragon Institute of Massachusetts General Hospital, Massachusetts Institute of Technology and Harvard University, Boston, Massachusetts, USA; d Center for Virology and Vaccine Research, Beth Israel Deaconess Medical Center, Boston, Massachusetts, USA; e Division of Infection and Immunity, University College London, London, United Kingdom; Emory University

**Keywords:** acute HIV-1 infection, HIV-1 envelope, HIV-1 intraparticipant diversity, HIV-1 subtype C, longitudinal HIV-1 diversity, sensitivity to bNAbs, bNAbs

## Abstract

Broadly neutralizing antibodies (bNAbs) for HIV-1 prevention or cure strategies must inhibit transmitted/founder and reservoir viruses. Establishing sensitivity of circulating viruses to bNAbs and genetic patterns affecting neutralization variability may guide rational bNAbs selection for clinical development. We analyzed 326 single *env* genomes from nine individuals followed longitudinally following acute HIV-1 infection, with samples collected at ~1 week after the first detection of plasma viremia; 300 to 1,709 days postinfection but prior to initiating antiretroviral therapy (ART) (median = 724 days); and ~1 year post ART initiation. Sequences were assessed for phylogenetic relatedness, potential N- and O-linked glycosylation, and variable loop lengths (V1 to V5). A total of 43 *env* amplicons (median = 3 per patient per time point) were cloned into an expression vector and the TZM-bl assay was used to assess the neutralization profiles of 15 bNAbs targeting the CD4 binding site, V1/V2 region, V3 supersite, MPER, gp120/gp41 interface, and fusion peptide. At 1 μg/mL, the neutralization breadths were as follows: VRC07-LS and N6.LS (100%), VRC01 (86%), PGT151 (81%), 10-1074 and PGT121 (80%), and less than 70% for 10E8, 3BNC117, CAP256.VRC26, 4E10, PGDM1400, and N123-VRC34.01. Features associated with low sensitivity to V1/V2 and V3 bNAbs were higher potential glycosylation sites and/or relatively longer V1 and V4 domains, including known “signature” mutations. The study shows significant variability in the breadth and potency of bNAbs against circulating HIV-1 subtype C envelopes. VRC07-LS, N6.LS, VRC01, PGT151, 10-1074, and PGT121 display broad activity against subtype C variants, and major determinants of sensitivity to most bNAbs were within the V1/V4 domains.

**IMPORTANCE** Broadly neutralizing antibodies (bNAbs) have potential clinical utility in HIV-1 prevention and cure strategies. However, bNAbs target diverse epitopes on the HIV-1 envelope and the virus may evolve to evade immune responses. It is therefore important to identify antibodies with broad activity in high prevalence settings, as well as the genetic patterns that may lead to neutralization escape. We investigated 15 bNAbs with diverse biophysical properties that target six epitopes of the HIV-1 Env glycoprotein for their ability to inhibit viruses that initiated infection, viruses circulating in plasma at chronic infection before antiretroviral treatment (ART), or viruses that were archived in the reservoir during ART in subtype C infected individuals in South Africa, a high burden country. We identify the antibodies most likely to be effective for clinical use in this setting and describe mutational patterns associated with neutralization escape from these antibodies.

## INTRODUCTION

Human immunodeficiency virus type 1 (HIV-1)-specific broadly neutralizing antibodies (bNAbs) may facilitate new approaches for HIV-1 prevention, treatment, or even cure (1, 2). bNAbs can neutralize a variety of circulating HIV-1 strains and are naturally produced in a small set of infected individuals (3). Innovative research has led to the development of bNAbs with improved features such as longer half-lives, favorable safety profiles (2, 4, 5), and potential to engage other components of the host immune response (6, 7). These bNAbs primarily target the trimeric HIV-1 envelope (Env) glycoprotein heterodimers (8, 9). Six bNAb categories based on recognition of the following distinct and largely conserved epitopes on the Env glycoprotein have been characterized: the V1/V2 loop at the trimer apex, the V3 region, the CD4 binding site (CD4bs), the membrane-proximal external region (MPER), the gp120/gp41 interface, and the fusion peptide (3, 10–13). Diverse antibodies provide insights for rational vaccine and immunogen design because they have different biophysical properties that may translate into variable potency or breadth, even when they target the same epitope (14, 15). Recent studies of passive administration of bNAbs in animal models and in a human clinical trial have provided proof of concept that they can protect against HIV-1 infection (16–19). Moreover, in HIV-1-infected individuals, passively administered bNAbs can suppress virus replication (20, 21).

Although bNAbs offer hope for new immune-based HIV prevention and treatment approaches, the Env as an immune target presents unprecedented challenges due its enormous diversity, partly driven by immune and other selection pressures (22–25). HIV is characterized by rapid evolution within and among infected individuals (26); which increases during chronic infection (27–29). This diversity presents a challenge for passive immunization strategies, as bNAbs targeting common Env epitopes would need to neutralize the transmitted/founder (TF) viruses for prophylaxis (5, 30), as well as the variants that emerge in the face of immune pressure and antiretroviral therapy (ART) for therapeutic or cure applications (31, 32). Considering the diverse bNAb biophysical properties and virus diversity, identification and characterization of the most suitable bNAbs for potential clinical use is a critical scientific goal. It should be noted that the forces that drive viral evolution have not been fully understood and characterized. Understanding these forces could have implications for the choice of bNAbs for prevention versus treatment/cure. Since the Env protein is exposed on the outer surface of HIV-1, it is the main target for neutralizing antibodies (33). Moreover, changes in particular regions of Env, including the five variable (V1 to V5) loops, may impact on sensitivity to bNAbs (34, 35). Therefore, it is important to characterize these changes not only to understand processes that lead to neutralization escape, but also to develop immunogens that can prevent escape. Escape mechanisms and pathways remain incompletely understood and they could open strategies for novel interventions to prevent escape or increase antibody potency and breadth. In addition, longitudinal studies that aim to assess *env* variant genetic diversity and neutralization sensitivity, as well as analyze key Env features in viruses derived from circulation and reservoirs before and after ART initiation during acute or chronic HIV-1 infection are lacking. We hypothesized that bNAbs targeting different epitopes will display heterogeneity in breadth and potency against circulating and reservoir-derived pseudotyped subtype C Envs. Specifically, we hypothesized that patterns of neutralization will differ among (i) TF viruses; (ii) viruses circulating in plasma (before treatment [BT]) and proviruses derived from peripheral blood and lymph node (LN) cells before ART initiation at chronic infection; and (iii) viruses archived in the peripheral blood (after treatment [AT]) or LN reservoirs following suppressive ART. In addition, we postulated that reduced neutralization sensitivity will be associated with distinct signature Env amino acid patterns such as differences in loop lengths, numbers of potential N-linked glycosylation sites and serine/threonine counts.

To test these hypotheses, we characterized the genetic plasticity of subtype C Envs among (i) TF viruses, (ii) viruses circulating in plasma, peripheral blood, and lymph nodes before ART initiation at chronic infection stage, and (iii) viruses archived in peripheral blood and lymph node reservoirs following ~1 year of suppressive ART in patients followed longitudinally following acute HIV infection. Furthermore, we investigated the impact of infection phase and compartment and genetic variability on the breadth/potency of bNAbs targeting CD4bs (VRC01, VRC07-LS, 3BNC117, and N6.LS); V1/V2 region (PGDM1400 and CAP256.VRC26.25); V3 supersite (PGT121, 10-1074, PGT128, PGT135, and 2G12); MPER (10E8 and 4E10); gp120/gp41 interface (PGT151); and fusion peptide (N123-VRC34.01).

## RESULTS

### Longitudinal analysis of intra- and interparticipant phylogenetic relatedness of clade C envelopes.

**FIG 1:**
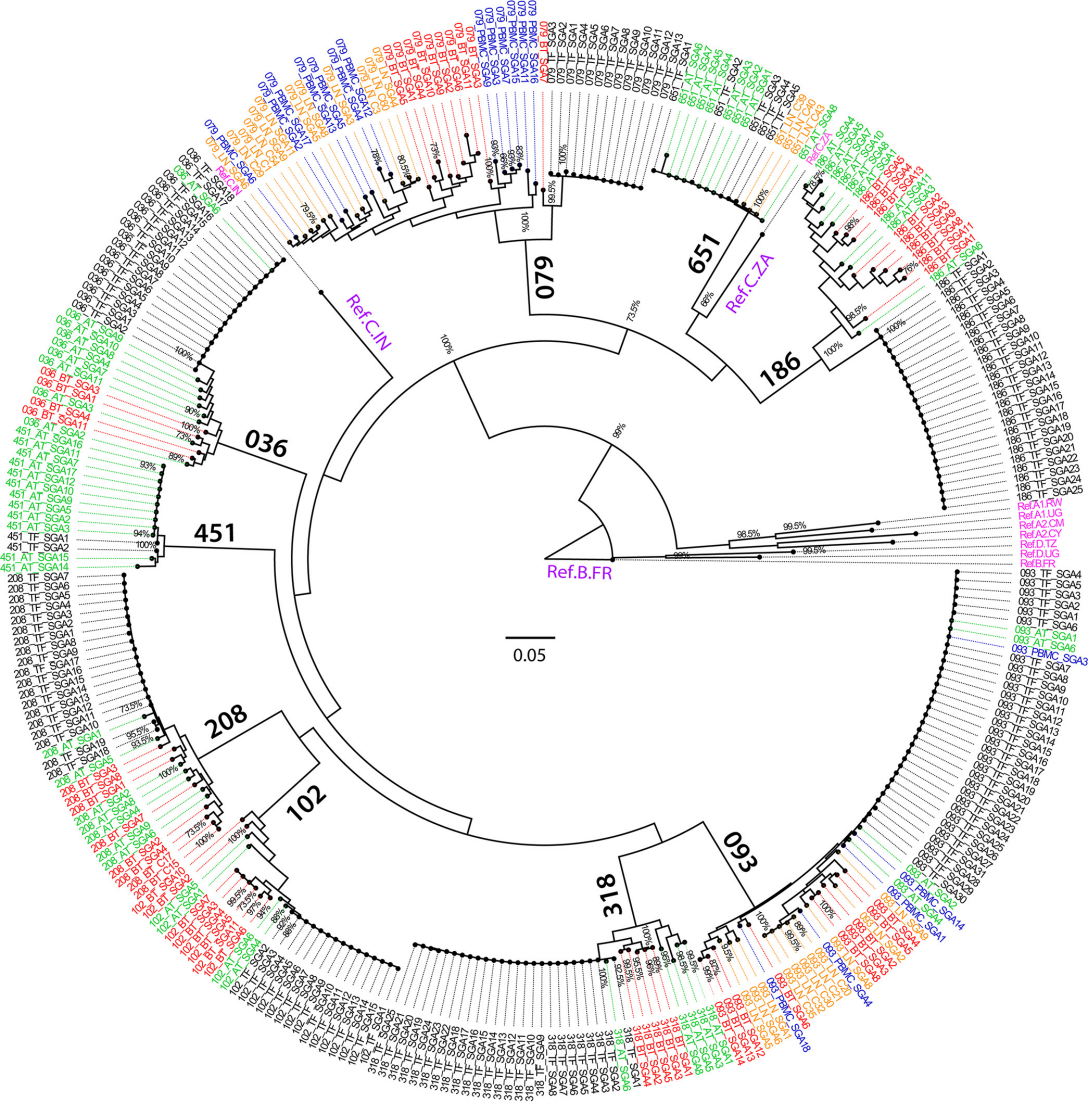
Maximum likelihood phylogenetic tree of 317 full-length subtype C HIV Env amino acid sequences. Transmitter/founder (TF) sequences are shown with black labels, chronic sequences (BT, before treatment) are denoted by red labels, while PBMC reservoir sequences (AT, ~1 year after treatment) are denoted by green labels. Lymph node (LN)-derived sequences for participants 079, 093, and 651 are denoted by orange labels, whereas their matching time point PBMC-derived sequences are denoted by blue labels. The branch length is drawn to scale to assess the relatedness between different sequences, with branch lengths measured in the number of substitutions per site. Model/method, Jones-Taylor-Thornton (JTT) model; Test of phylogeny, bootstrap method; No. of bootstrap replications, 200 (86–88). Reference sequences (denoted in pink) included: (Ref. A1.RW accession number BAF31340.1); (Ref. A1.UG accession number BAF31412.1); (Ref. A2.CM accession number ADA83368.1); (Ref. A2.CY accession number AAK65976.1); (Ref. D.TZ accession number AAQ98188.1); (Ref. D.UG accession number AAC97570.1); and (Ref. B.FR accession number NP057856.1); (Ref. C.ZA accession number AAV41353.1) and (Ref. C.IN accession number AAD12085.1).

We examined the inter- and intraparticipant phylogenetic relatedness of *env* sequences from the nine participants. Each participant was sampled at 3 time points (TF virus; ~1 week post detection of plasma viremia; time point just prior to ART initiation [median = 724 days] and ~1 year post ART initiation). In addition, three participants 079, 093 and 651, were also sampled from the lymph node and the PBMC compartments at matching time points. In total, we generated a total of 326 full-length gp160 sequences by single-genome amplification (SGA) (median = 9 sequences per time point). Following exclusion of sequences with multiple stop codons the analysis involved a total of 317 gp160 amino acid sequences, which were analyzed for phylogenetic relatedness (Fig. 1). Individual trees for each participant are also shown in Fig. S1 in the supplemental material.

As expected, all sequences clustered with South African (Ref. C.ZA accession number AAV41353.1) and Indian (Ref. C.IN accession number AAD12085.1) clade C reference sequences and clustered distinctly away from nonclade C reference sequences. All the studied participants appeared to be single variant transmissions (identical or near-identical clusters of the TF sequences). For two participants (451 and 651) who initiated ART within 1 week of positive HIV-1 RNA plasma detection, the variants isolated in the reservoir compartments (PBMCs and lymph node) clustered closely with their corresponding TF variants. Among the six participants (036, 093, 102, 186, 208, and 318) who initiated ART at the chronic stage of infection, variants that were circulating in plasma before ART initiation clustered independently of the TF variants. Moreover, the variants that were circulating in the PBMC reservoir after ~1 year of suppressive ART clustered independently of the TF variants. In addition, for participant 093 who was also sampled from the LN under suppressive ART, the LN variants clustered independently of the TF variants. Overall, there was intermixing of the variants isolated from plasma at chronic stage of infection before ART initiation and the variants in the PBMC/LN following ~1 year of suppressive ART. Participant 079, who was ART-naive throughout the duration of the study, also exhibited viral diversification over time, with the TF variants clustering independently of the variants in the plasma, PBMC, and LN at chronic stage of infection. Interestingly, among participants 036, 093, 208, and 318, there were some variants resembling the TF that were still identifiable in the PBMC compartment following ~1 year of suppressive ART. Taken together, this analysis is consistent with the consensus in the field that there is a stringent genetic bottleneck governing transmission and early ART curtails intrahost viral diversification. In contrast, when ART initiation is delayed until the chronic stage of infection, this creates an opportunity for intrahost viral diversification and the variants in the reservoir generally resemble those that were circulating prior to ART initiation.

### Intra- and interparticipant longitudinal assessment of variable domains (V1 to V5).

**FIG 2:**
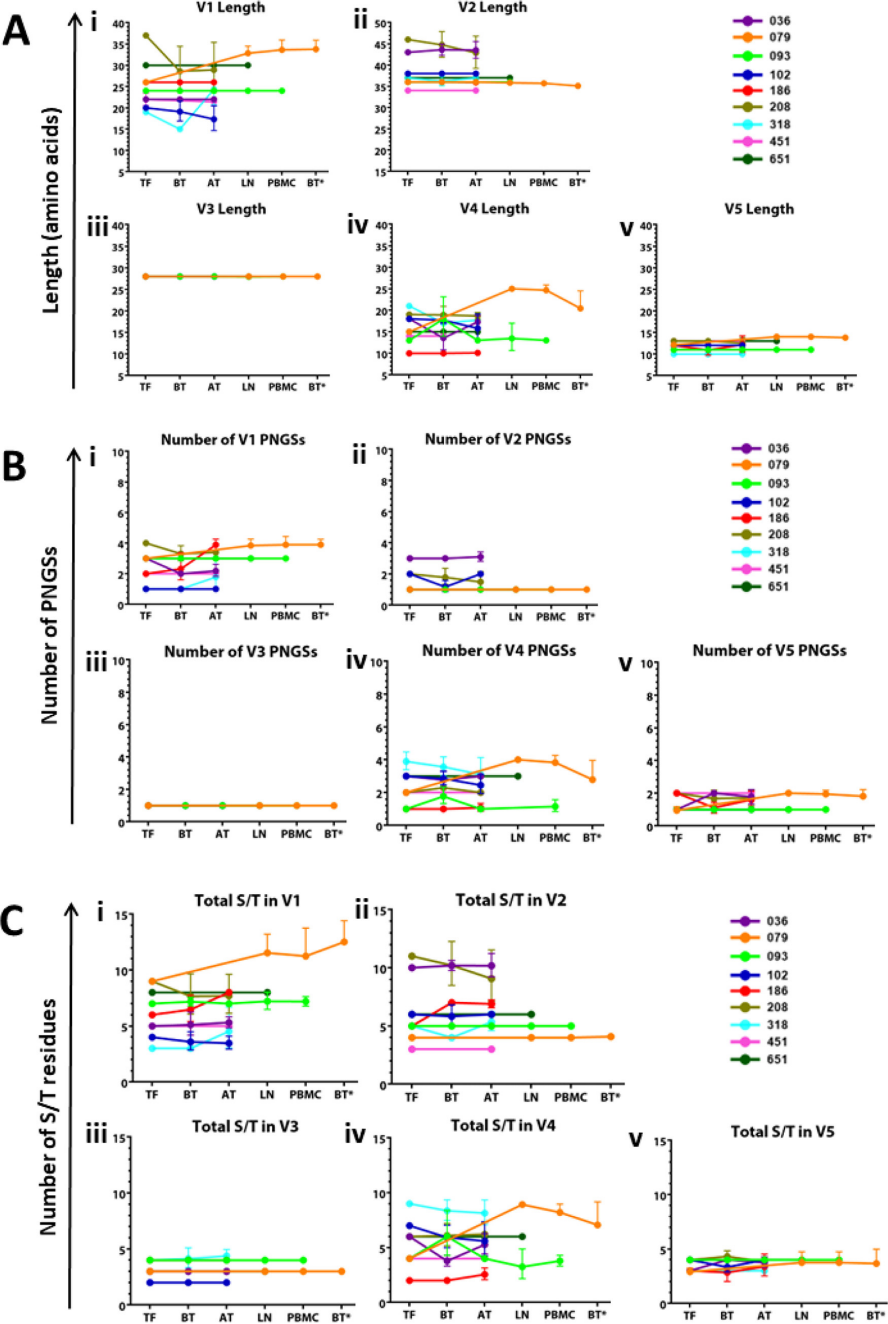
Line graphs showing distribution of the V1 to V5 loop characteristics over time. (A) V1 to V5 length; (B) V1 to V5 PNGSs; and (C) V1 to V5 S/T counts. Each color-coded line represents a different participant. The geometric mean distribution of each parameter is represented as a dot for each time point. *y* axis, subregion variable loop parameter; *x* axis, sampled compartment and the relative days post observed plasma viremia increase from left to right (TF, transmitted/founder; BT, before treatment; AT, after treatment; LN, lymph node; and PBMC, sequences from PMBC at time point matching lymph node excision). The asterisk (*) denotes sequences obtained from participant 079 only, who remained ART naive throughout the study. Time points TF, BT, AT, LN, and PBMC differ from participant to participant, please see the exact time points in Table 2. Logo plots showing longitudinal intraparticipant V1 and V4 sequence variation are shown in Fig.S3 and S4.

To further characterize the *env* sequence diversity among the nine studied participants, we next investigated the evolution of sequences within variable domains (V1 to V5). We analyzed the HIV-1 *env* variants immediately following infection (within 1 week) in all nine participants approximately 300 to 1,709 days postinfection, but prior to initiating ART (median = 724 days postinfection) in 7/9 participants, and approximately 1 year after initiating ART in 8/9 participants. HIV-1 variants were sampled from plasma prior to initiating ART (before treatment [BT]); however, in some participants who later commenced therapy during the chronic stage, as well as those who initiated ART at the acute stage, the variants were sampled from PBMCs approximately 1 year after initiating ART (after treatment [AT]). Lymph node variants from participants 651 and 093 were also analyzed while on suppressive ART at 468 days and 1,044 days post detection of plasma viremia, respectively. We also analyzed LN variants from the ART-naive participant 079 at 1,296 days post detection of plasma viremia. In addition, we also analyzed the variants in PBMC and the LN at matched time points for 093 and 079, except for 651. The sequence analysis concentrated on three characteristics: domain lengths, potential N-linked glycosylation sites (PNGSs), and total serine/threonine (S/T) residue counts (Fig. 2).

### Intra- and interparticipant longitudinal assessment of clade C envelopes’ variable domain length.

Previous studies have noted alterations in the lengths of variable loops within gp120 to be among some of the mechanisms that HIV-1 uses to escape neutralizing antibody mediated humoral responses (36, 37). Therefore, we assessed the intraparticipant lengths of variable regions between TF sequences and those derived from subsequent time points (before and after ART initiation). As shown in Fig. 2A (i), the V1 loop lengths for all studied participants ranged between 15 and 37 amino acids (AAs). For participants 079 and 318, the V1 lengths increased from (25 to 35) and (20 to 24) AAs over 1,536 and 1,417 days post onset of plasma viremia (DPOPV), respectively. The V1 lengths for participants 102 and 208 decreased from (20 to 15) and (37 to 29) AAs over 1,095 and 867 DPOPV, respectively. Noteworthy is that participants 036, 093, 186, 451, and 651 had constant V1 lengths at 22, 24, 26, 22, and 30 AAs over 1,102, 1,044, 745, 318 and 468 DPOPV, respectively. As shown in Fig. 2A (ii), the V2 loop lengths for all studied participants ranged between 34 and 48 AAs. Whereas the majority of intraparticipant V2 domain lengths were constant over time, participant 036 registered an increase of AAs (43 to 46), while participant 208 showed a decrease from 46 to 43 AAs. Interestingly the V3 domain length was conserved at 28 AAs for all participants (Fig. 2A [iii]). As shown in Fig. 2A (iv), the V4 loop lengths for most studied participants was dynamic, ranging between 10 and 25 AAs. For participants 079 and 318, the V4 lengths increased from 15 to 25 and decreased from 21 to 18 AAs over 1,536 and 1,417 DPOPV, respectively. The V4 lengths for participants 036 and 093 fluctuated between 18 and 10 and 13 and 23 AAs over 1,102 and 1,044 DPOPV, respectively. Noteworthy is that the V4 length for participant 102 decreased from (18 to 13) AAs over 1,095 DPOPV. Participants 186, 208, 451, and 651 had constant V4 lengths at 10, 19, 14, and 15 AAs over 745, 867, 318 and 468 DPOPV, respectively (Fig. 2A [iv]). Interestingly, the intraparticipant V5 domain lengths were largely constant with an overall range between 10 and 13 AAs. However, a subtle increase and fluctuation (within 1 AA) was observed over time for participants 079 and 186, respectively (Fig. 2A [v]). Taken together, these results reveal the high genetic plasticity of the V1 and V4 domain lengths (increase, decrease, or constant) over time following acute and chronic HIV-1 infection. In contrast, the intraparticipant V2, V3, and V5 domain lengths are predominantly conserved over time during acute and chronic HIV-1 infection.

### Intra- and interparticipant longitudinal assessment of clade C envelopes’ variable domain potential N-linked glycosylation sites.

The HIV-1 gp120 is highly glycosylated, with an estimated 50% of its total mass comprising host-derived N-linked glycans. Earlier reports have noted alterations in the PNGSs of variable loops within gp120 to be among drivers of neutralizing antibody escape in HIV-1 (37, 38). To assess the longitudinal alterations in PNGSs, we computed the intra participant PNGSs of variable regions between TF sequences and subsequent chronic time points (before and after ART initiation). As shown in Fig. 2B (i), the V1 loop for all studied participants ranged between 1 and 4 PNGSs. For participants 079, 186, and 318, the V1 PNGSs increased from 3 to 4, 2 to 4, and 1 to 2 over 1,536, 745, and 1,417 DPOPV, respectively. In participants 036, and 208, the V1 PNGSs decreased from 3 to 2, and 4 to 3 over 1,102, and 867 DPOPV, respectively. Participants 093, 102, 451, and 651 had constant V1 PNGSs of 3, 1, 2, and 2 over 1,044, 1,095, 318, and 468 DPOPV, respectively. Interestingly, the intraparticipant V2 domain PNGSs were largely constant with an overall range between 1 and 3 PNGSs, except for participant 208, which showed a decrease in PNGSs from 2 to 1 over 867 DPOPV (Fig. 2B [ii]). Moreover, the intraparticipant V3 domain PNGSs were all constant with 1 PNGS over time (Fig. 2B [iii]). As shown in Fig. 2B (iv), the V4 loop PNGSs for most studied participants was dynamic, ranging between 1 and 4 PNGSs. For participant 079, the V4 PNGSs increased from 2 to 4 over 1,536 DPOPV; while for participant 318, the V4 PNGSs decreased from 4 to 3 over 1,417 DPOPV. Fluctuations in PNGSs were registered between 1 and 2 PNGSs in participant 093 over 1,044 DPOPV. Overall, the V5 domain for all studied participants ranged between 1 and 2 PNGSs. For participants 036 and 079, the V5 PNGSs increased from 1 to 2 over 1,102 and 1,536 DPOPV, respectively (Fig. 2B [v]). In summary, these results show a variable pattern (increase, decrease, and constant) for V1 and V4 domain PNGSs over time following acute and chronic HIV-1 infection. However, the intraparticipant V2, V3, and V5 domain PNGSs are predominantly conserved over time during acute and chronic HIV-1 infection.

### Longitudinal variation in intra- and interparticipant clade C Env variable domain total S/T counts.

The S/T residues are potential hot spots for O-linked glycosylation (39); therefore, the computation of total S/T count is a crude estimation of potential for O-linked glycosylation. Until recently (40), several studies have reported no evidence for O-linked glycosylation of HIV-1 Env proteins (41). To assess the longitudinal alterations in S/T counts, we computed the intraparticipant total S/T counts of variable regions between TF sequences and subsequent time points (before and after ART initiation). As shown in Fig. 2C (i), the V1 loop for all studied participants had S/T counts ranging between 3 and 14. For participants 079, 186, and 318, the V1 increased its S/T counts from 9 to 14, 6 to 8, and 3 to 4 over 1,536, 745, and 1,417 DPOPV, respectively. In participants 102 and 208, the V1 S/T counts decreased from 4 to 3 and 9 to 8 over 1,095 and 867 DPOPV, respectively. Participants 036, 093, 451, and 651 had constant V1 S/T counts of 5, 7, 5, and 8 over 1,102, 1,044, 318, and 468 DPOPV, respectively. As shown in Fig. 2C (ii), the V2 loop S/T counts for all studied participants ranged between 3 and 12. Interestingly, the intraparticipant V2 domain S/T counts were largely constant, except for participant 208, which had its V2 domain S/T counts decreasing from 11 to 9 over 867 DPOPV, while participant 186 had V2 domain S/T counts increasing from 5 to 7 over 867 DPOPV. Notably, the V2 domain S/T counts of 318 fluctuated between 5 and 4 over 1,417 DPOPV. Moreover, the intraparticipant V3 domain S/T counts were all constant ranging between 2 and 4 over time (Fig. 2C [iii]). As shown in Fig. 2C (iv), the V4 loop S/T counts for most studied participants were dynamic, ranging between 2 and 9. For participants 079 and 186 the V4 S/T counts increased from 4 to 9 and 2 to 3 over 1,536 and 745 DPOPV, respectively. The V4 S/T counts for participants 093, 102, and 318 decreased from 4 to 3, 7 to 6, and 9 to 8 over 1,044, 1,095, and 1,417 DPOPV, respectively. Participants 451, and 651 had constant V4 S/T counts at 4 and 6 over 318 and 468 DPOPV, respectively. Interestingly, the intraparticipant V5 domain S/T counts were largely constant with an overall range between 2 and 5 S/T counts (Fig. 2C [v]). The data reveal that the V1 and V4 domain S/T counts can rise, decrease, or remain constant over time during acute and chronic HIV-1 infection. However, the intraparticipant V2, V3, and V5 domain S/T counts are mostly conserved.

### Investigation of epitope-targeted neutralization susceptibility of longitudinal intra- and interparticipant clade C HIV-1 pseudotyped envelopes.

To assess longitudinal alterations in sensitivity to key broadly neutralizing antibodies, we cloned representative and phylogenetically distinct single genome sequences (*n* = 67; median = 3 per time point per participant) per time point for TZM-bl HIV neutralization antibody assays. The selection criteria of sequences for cloning were based on similarity to consensus of the sampled time point per participant. For more diverse time points, clones were also selected based on furthest distance from the consensus to ensure representative sampling. However, based on the 50% tissue culture infective dose (TCID_50_) assessed through titration of the pseudovirus stocks produced through transfections in 293T cells, only 43 out of the 62 nontransmitted founder clones (69%) were functional (Table 1).

**TABLE 1:** Summary of the HIV-1 *env* clones generated and assessment of their ability to yield functional pseudotyped envelopes^*a*^

PID	SGA clone ID	Compartment	DPOPV	Treatment status	Days PTI	Functional clone
036	036 TF	Plasma	1	No ART	−724	Yes
	036 BT SGA1	Plasma	665	No ART	−59	No
	036 BT SGA4	Plasma	665	No ART	−59	Yes
	036 BT SGA11	Plasma	665	No ART	−59	No
	036 AT SGA3	PBMC	1,102	On ART	366	No
	036 AT SGA4	PBMC	1,102	On ART	366	No
	036 AT SGA6	PBMC	1,102	On ART	366	No
079	079 TF	Plasma	1	No ART	−1,709	Yes
	079 LN SGA3	Lymph node	1,296	No ART	−413	Yes
	079 LN SGA6	Lymph node	1,296	No ART	−413	No
	079 LN SGA9	Lymph node	1,296	No ART	−413	Yes
	079 PBMC SGA7	PBMC	1,295	No ART	−413	Yes
	079 PBMC SGA9	PBMC	1,296	No ART	−413	Yes
	079 PBMC SGA10	PBMC	1,296	No ART	−413	No
	079 PBMC SGA12	PBMC	1,296	No ART	−413	Yes
	079 PBMC SGA17	PBMC	1,296	No ART	−413	Yes
	079 BT SGA3	Plasma	1,536	No ART	−173	Yes
	079 BT SGA4	Plasma	1,536	No ART	−173	Yes
	079 BT SGA7	Plasma	1,536	No ART	−173	No
093	093_TF	Plasma	1	No ART	−310	Yes
	093 BT SGA3	Plasma	252	No ART	−58	Yes
	093 BT SGA4	Plasma	252	No ART	−58	No
	093 BT SGA13	Plasma	252	No ART	−58	Yes
	093 AT SGA1	PBMC	693	On ART	372	Yes
	093 AT SGA4	PBMC	693	On ART	372	No
	093 LN SGA6	Lymph node	1,044	On ART	660	Yes
	093 LN SGA8	Lymph node	1,044	On ART	660	Yes
	093 LN SGA9	Lymph node	1,044	On ART	660	Yes
	093 PBMC SGA1	PBMC	1,044	On ART	660	No
	093 PBMC SGA4	PBMC	1,044	On ART	660	Yes
	093 PBMC SGA14	PBMC	1,044	On ART	660	Yes
102	102 BT SGA2	Plasma	771	No ART	−38	No
	102 BT SGA3	Plasma	771	No ART	−38	Yes
	102 BT SGA6	Plasma	771	No ART	−38	Yes
	102 AT SGA1	PBMC	1,095	On ART	276	Yes
	102 AT SGA5	PBMC	1,095	On ART	276	Yes
	102 AT SGA6	PBMC	1,095	On ART	276	No
186	186 TF	Plasma	1	No ART	−347	Yes
	186 BT SGA1	Plasma	333	No ART	−14	Yes
	186 BT SGA2	Plasma	333	No ART	−14	No
	186 AT SGA1	PBMC	745	On ART	387	Yes
	186 AT SGA5	PBMC	745	On ART	387	Yes
	186 AT SGA9	PBMC	745	On ART	387	No
208	208 TF	Plasma	1	No ART	−427	Yes
	208 BT SGA1	Plasma	413	No ART	−14	No
	208 BT SGA3	Plasma	413	No ART	−14	No
	208 BT SGA10	Plasma	413	No ART	−14	Yes
	208 AT SGA4	PBMC	867	On ART	426	No
	208 AT SGA7	PBMC	867	On ART	426	Yes
318	318TF	Plasma	1	No ART	−1,059	Yes
	318 BT SGA1	Plasma	990	No ART	−69	No
	318 BT SGA4	Plasma	990	No ART	−69	Yes
	318 BT SGA5	Plasma	990	No ART	−69	Yes
	318 AT SGA1	PBMC	1,417	On ART	348	Yes
	318 AT SGA6	PBMC	1,417	On ART	348	No
	318 AT SGA8	PBMC	1,417	On ART	348	Yes
451	451 TF	Plasma	1	No ART	0	Yes
	451 AT SGA4	PBMC	318	On ART	318	No
	451 AT SGA5	PBMC	318	On ART	318	Yes
	451 AT SGA15	PBMC	318	On ART	318	No
651	651 AT SGA2	PBMC	327	On ART	327	Yes
	651 AT SGA5	PBMC	327	On ART	327	Yes
	651 AT SGA6	PBMC	327	On ART	327	No
	651 AT SGA8	PBMC	327	On ART	327	Yes
	651 c39	Lymph node	468	On ART	468	No
	651 c40	Lymph node	468	On ART	468	Yes
	651 c43	Lymph node	468	On ART	468	Yes

Total clones (non-TF)						62
Total functional clones						43
Functionality (%)						69%

a TF, transmitted/founder; BT, plasma before ART at chronic; AT, PBMC post ~1 year on ART; LN, lymph node derived; PBMC, derived from PBMC at time point matching lymph node; DPOPV, days post first detection of plasma viremia; Days PTI, days post treatment initiation.

**FIG 3:**
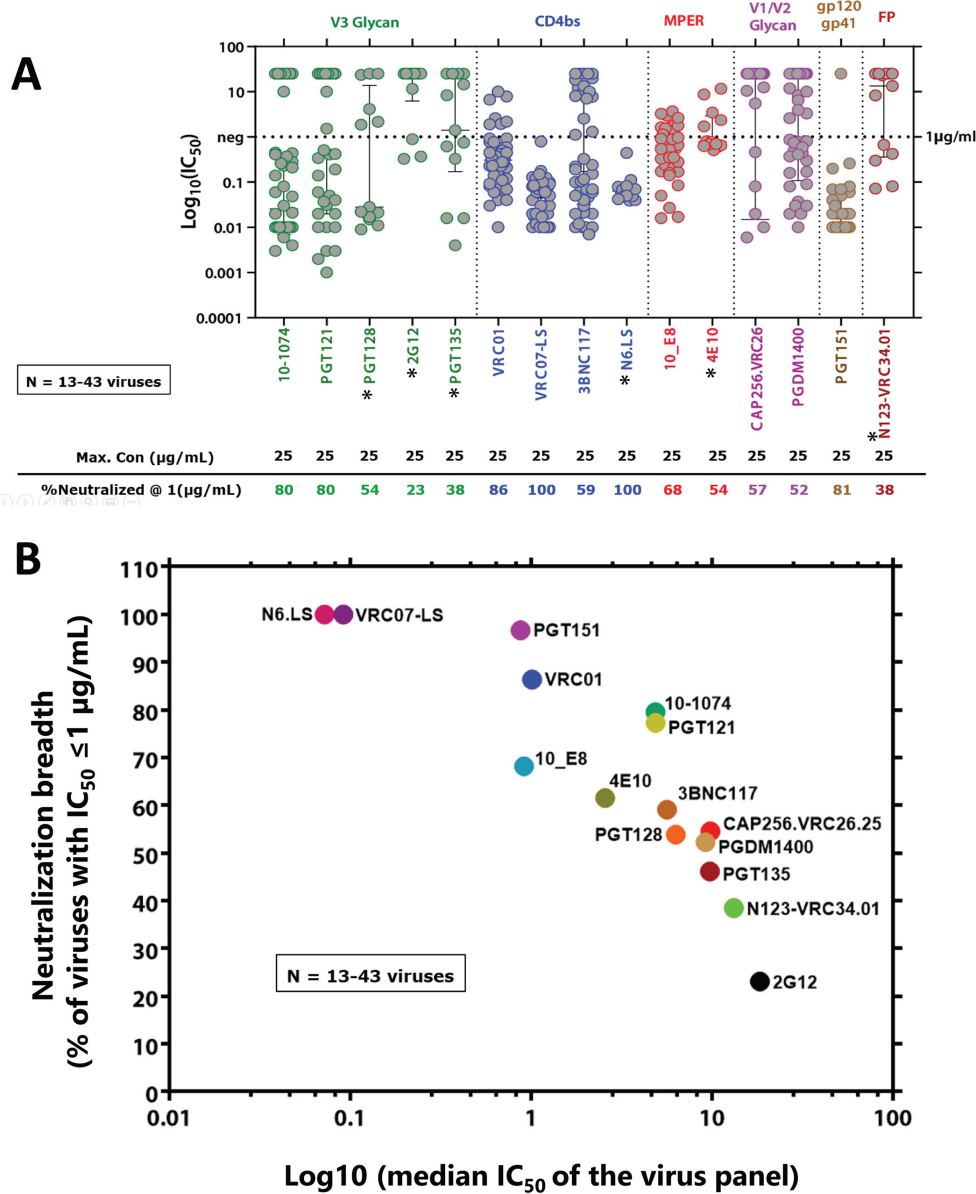
Epitope-targeted neutralizing antibody-phenotyping of acute and chronic phase HIV-1 subtype C envelopes. (A) Neutralization data are presented as scatterplots of IC_50_ titers (μg/mL) in which each virus is represented by an individual circle. Antibodies targeting each epitope are color-coded. The highest concentration tested for each bNAb, and the percentage of viruses (*n* = 43) neutralized are indicated. Solid black bars represent median titers and their interquartile ranges. The asterisks (*) adjacent to each bNAb indicate the corresponding number of viruses (*n* = 13) tested against that antibody; The rest of the bNAbs were tested against 43 viruses, apart from PGT151, which was tested against 30 viruses. (B) Neutralization breadth (% of viruses with IC_50_ ≤1 μg/mL) and potency of each tested bNAb (median IC_50_ of the subtype C virus panel [*n* = 13 to 43] against each bNAb). The neutralization was assessed by a TZM-bl pseudovirus assay. Each circle represents the neutralization breadth at a given median IC_50_ (μg/mL) of the virus panel. Each color-coded circle represents neutralization by a respective bNAb.

We then proceeded to examine the sensitivity of the 43 functional clones against a panel of 15 bNAbs targeting CD4bs (VRC01, VRC07-LS, 3BNC117, and N6.LS), the V1/V2 region (PGDM1400 and CAP256.VRC26.25) and V3 supersite (10-1074, PGT121, PGT128, 2G12, and PGT135), MPER (10E8 and 4E10), gp120/gp41 interface (PGT151), and fusion peptide (FP) (N123-VRC34.01). As shown in Fig. 3A, the maximum concentration of each tested antibody was 25 μg/mL. For neutralization breadth, we used an IC_50_ threshold of ≤1 μg/mL, which was the lower limit of range of bNAb serum concentrations in HIV-1 infected individuals who were administered a single dose of 1 to 30 mg/kg of 3BNC117 (42). Among the tested V3 glycan-targeting antibodies, 10-1074 and PGT121 had 80% breadth at 1 μg/mL against the tested pseudoviruses, while PGT128, 2G12, and PGT135 had breadths of 54%, 23%, and 38%, respectively. The profiles of the CD4bs-targeting antibodies indicated that at 1 μg/mL, VRC01, VRC07-LS, 3BNC117, and N6.LS had breadths of 86%, 100%, 59%, and 100%, respectively. At 1 μg/mL, the MPER-targeting antibodies 10E8 and 4E10 had breadths of 68% and 54%, respectively, while the V1/V2-targeting antibodies CAP256.VRC26 and PGDM1400 had breadths of 57% and 52%, respectively. Lastly, PGT151 and N123-VRC34.01, which target the gp120/gp41 interface and the fusion peptide, respectively, had a breadth of 81% and 38%, respectively. Taken together, these data (summarized in Fig. 3B) indicate that the best performing antibodies targeting HIV-1 subtype C were as follows: VRC07-LS and N6.LS (both with 100% breadth) for CD4bs-targeting antibodies; 10-1074 and PGT121 (both with 80% breadth) for V3-targeting antibodies; 10E8 (68% breadth) for an MPER-targeting antibody; PGT151 (81% breadth) for the gp12/gp41 interface. Moreover, it appeared that the V1/V2-targeting CAP256.VRC26 and PGDM1400 (57% and 52% breadth, respectively), alongside the FP-targeting N123-VRC34.01 (38% breadth) were moderately to weakly neutralizing. These data suggest that future studies may need to identify more broadly neutralizing antibodies targeting V1/V2 and FP epitopes within subtype C Envs.

### Evidence of heterogeneity in neutralization sensitivity among TF viruses, circulating plasma, and reservoir-derived variants against some bNAb classes.

**FIG 4:**
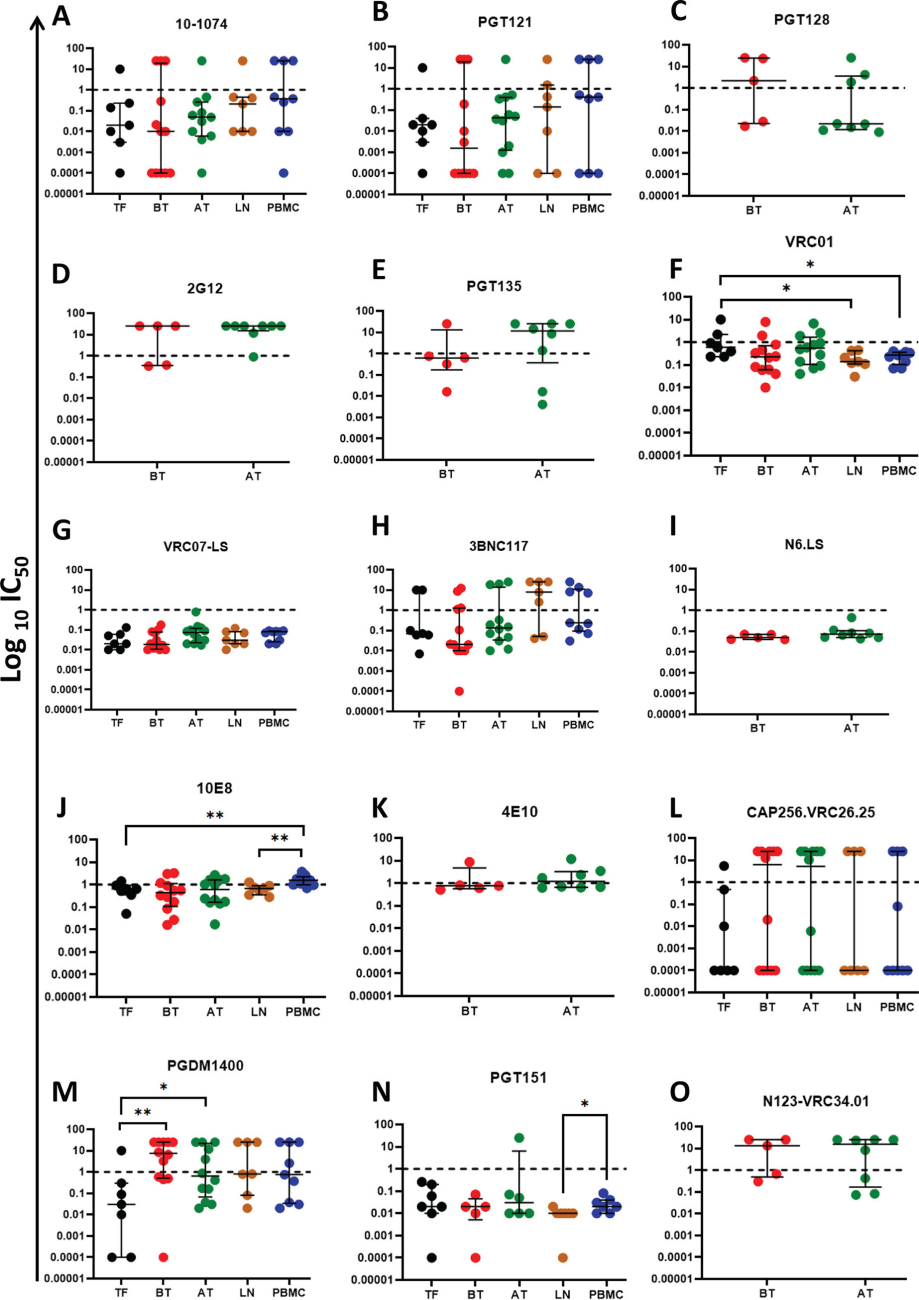
Longitudinal epitope-targeted neutralization IC_50_ titers for each tested bNAb. (A to E) V3-targeting antibodies: 10-1074, PGT121, PGT128, 2G12, and PGT135. (F to I) CD4bs-targeting antibodies: VRC01, VRC07-LS, 3BNC117, and N6.LS. (J and K) MPER-targeting antibodies: 10E8 and 4E10. (L and M) V1/V2-targeting antibodies: CAP256.VRC26.25 and PGDM1400. (N) gp120/gp41 interface-targeting antibody: PGT151. (O) Fusion peptide-targeting antibody: N123-VRC34.01. Each dot represents each tested pseudovirus and each group represents the sampled time point/compartment. TF, transmitted founder viruses within 1 week of positive plasma viremia detection (black dots). BT, viruses circulating in plasma at chronic prior ART initiation (red dots). AT, viruses circulating in PBMC after ~1 year of ART (green dots). LN, viruses sampled from the lymph node (orange dots). PBMC, viruses sampled from the PBMC at a time point matching the lymph node (blue dots). Significant differences among groups of variants using the Mann-Whitney *unpaired t test* are indicated by asterisks. Nonsignificant, *P* > 0.05; *, *P* = 0.05 to 0.01; **, *P* = 0.01 to 0.001.

After establishing the overall breadth of each antibody against the panel of 43 viruses, we next wanted to assess whether there is heterogeneity in neutralization sensitivity between the following groups of variants: (i) TF viruses; (ii) viruses circulating in plasma prior to ART initiation during chronic stage; (iii) viruses archived in PBMC following ~1 year of ART; (iv) viruses derived from the lymph nodes; and (v) viruses derived from the PBMC at a time point that matches lymph nodes. As shown Fig. 4A to E, there was no statistically significant evidence of neutralization sensitivity distinction between TF viruses, plasma-derived viruses circulating during chronic infection, and reservoir-derived variants among V3-specific antibodies. A closer-examination of CD4bs-specific antibodies showed that there was statistically significant evidence of transmitted variants being more resistant to VRC01 than matching lymph node and PBMC variants. Moreover, VRC07-LS and N6.LS showed 100% breadth at 1 μg/mL against variants tested from each group (Fig. 4F–I). Investigation of MPER-specific bNAbs (Fig. 4J and K), showed statistically significant evidence of transmitted variants being more sensitive to 10E8 than the variants from matched PBMC and lymph node time points. Moreover, there was evidence that lymph node-derived variants were more sensitive to neutralization by 10E8 compared to PBMC variants at matched time points. However, for 4E10, there was no evidence of significant neutralization sensitivity distinction between variants circulating in plasma at chronic and variants in the PBMC reservoir after ~1 year of ART. Upon investigating V1/V2-specific bNAbs (Fig. 4L and M), there was statistically significant evidence of transmitted variants being more sensitive to PGDM1400 than the variants circulating at chronic before ART initiation as well as those archived in PBMC at ~1 year post ART initiation. Also noteworthy was the distinct pattern of sensitivity to neutralization by CAP256.VRC26.25, with variants displaying either high sensitivity or resistance, with no partial phenotype of sensitivity to this bNAb. For the gp120/gp41-specific bNAb PGT151, there was no statistically significant evidence of neutralization sensitivity distinction between transmitted, plasma-circulating at chronic, and PBMC-reservoir variants after ~1 year of ART. Nonetheless, there was evidence that lymph node-derived variants were more sensitive to neutralization by PGT151 compared to PBMC-derived variants at matched time points. However, for the fusion peptide-targeting antibody, N123-VRC34.01, there was no statistically significant evidence of neutralization sensitivity distinction between variants circulating in plasma at chronic and variants in the PBMC reservoir after ~1 year of ART. Taken together, VRC07-LS and N6.LS demonstrated the greatest potential for prevention and therapeutic application, considering that they exhibited 100% breadth and potency in cross-neutralizing TF, viruses circulating before treatment (BT), PBMC-derived viruses after treatment (AT), and variants from matched LN/PBMC time points. Additionally, these data imply that for some antibodies, distinct neutralization sensitivity patterns may arise between transmitted, plasma-circulating, and reservoir variants.

### Intraparticipant heterogeneity in neutralization sensitivity among transmitted, plasma-circulating, and reservoir variants against some bNAb classes.

Based on the categorized overall analysis of the TF, BT, AT, LN and PBMC variants, there was some evidence of neutralization sensitivity distinction among transmitted, plasma-circulating, and reservoir variants. Therefore, to mitigate intraindividual quasispecies bias in the interpretation of the data, we next longitudinally analyzed the nine participants individually (Fig. S2).

### V3 bNAbs.

**FIG 5:**
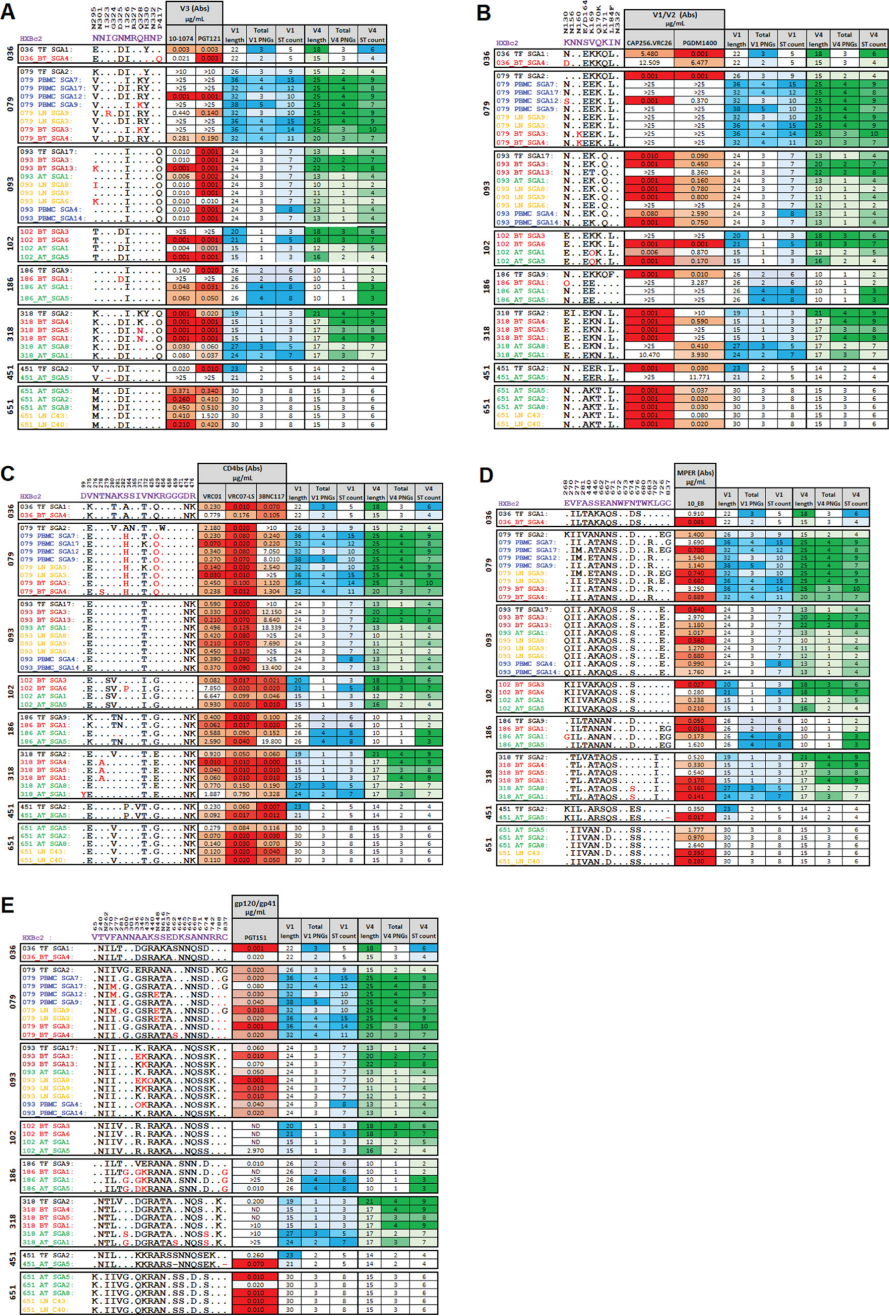
Signature mutations, V1/V4 sequence characteristics, and neutralization activity of antibodies against each epitope. (A) V3 antibodies; (B) V1/V2 antibodies; (C) CD4bs antibodies; (D) MPER antibody; and (E) gp120/gp41 interface antibody. In each panel, the first column shows the name of each tested representative clones, the second column shows their respective escape mutations from literature, denoted in vertical labels above the heatmap of each bNAb category (89) and in magenta, is the corresponding HXB2 AA numbering. The red and orange heatmaps (drawn per participant) show the neutralization activity (IC_50_ in μg/mL) of each antibody, where red is the most neutralizing and white least neutralizing. The blue heatmaps (drawn per participant) show the V1 loop characteristics (V1 length, total PNGs in V1 and total S/T counts in V1), where dark shade represents higher values and lighter shade represents the least values. The green heatmaps (drawn per participant) show the V4 loop characteristics (V4 length, total PNGs in V4 and total S/T counts in V4), where dark shade represents higher values and lighter shade represents the (lower values). TF, transmitted founder viruses within 1 week of positive plasma viremia detection. BT, viruses circulating in plasma at chronic prior ART initiation. AT, viruses circulating in PBMC after ~1 year of ART. LN, viruses sampled from the lymph node. PBMC, viruses sampled from the PBMC at a time point matching the lymph node.

As shown in Fig. S2A and B, among the tested V3-targeting antibodies 10-1074 and PGT121, similar IC_50_ profiles were observed longitudinally for participants 079, 093 and 651. Interestingly, the highest resistance to neutralization was consistently seen for variants from participant 079, which also had the highest V1 domain S/T counts for all its variants compared to any other participant (Fig. 2C [i]). For participants 036, 318, and 451, the TF had lower IC_50_ values for 10-1074 compared to corresponding variants sampled at later time points. A similar trend was observed against PGT121, except for participant 036 variants, which had consistent IC_50_ values over time. In contrast, the TF from participants 102 and 208 were less sensitive to both 10-1074 and PGT121 compared to the variants sampled from the same participants at later time points. In addition, as shown in Fig. 5A, we observed that the glycans that interact directly with V3 bNAbs at positions N332, N301, and N295 (43) that are associated with neutralization sensitivity to V3 bNAbs were longitudinally conserved in all participants. These data suggest that the intraparticipant neutralization sensitivity against V3 antibodies can vary over the course of infection.

### V1/V2 bNAbs.

As shown in Fig. S2C and D, the TF variants from 079, 208, 318, and 451 were more sensitive to CAP256.VRC26.25 compared to the variants sampled at later time points. Similarly, for PGDM1400, the TF variants from 036, 079, 186, 208, and 451, were more sensitive compared to the variants sampled at later time points. In contrast, the TF from participant 102 was more resistant to CAP256.VRC26.25 and PGDM1400 than corresponding variants sampled at later time points. Participant 093 had fluctuating IC_50_ values for the tested V1/V2 antibodies between transmission and later sampled time points. In addition, the heatmap illustration (Fig. 5B) suggests that the V1/V2 bNAbs contacting glycans at positions N156 and N160 did not guarantee sensitivity to CAP256.VRC26 and PGDM1400. Seven viruses had very similar distributions of IC_50_ titers to viruses that had the PNGS site for all V2 bNAbs tested. Nevertheless, the heatmap in Fig. 5B shows that when cross-sectionally and longitudinally examined, all the studied participants had largely conserved V1/V2 signature mutation profiles, despite varied IC_50_ titers in some instances (318_AT_SGA8 versus 318_AT_SGA1). The K169E/T mutation observed for participant 093 appeared to confer resistance to both CAP256.VRC26 and PGDM1400. Interestingly, the unusual V1 and V4 characteristics of select variants that were markedly of low sensitivity to the V3 bNAbs (in participants 079, 093, 102, 186, and 318) were high ST counts and/or relatively higher PNGSs and/or relatively longer lengths. Taken together, these data suggest that the intraparticipant neutralization sensitivity against the tested V1/V2 antibodies can follow a decreasing, increasing, fluctuating, or constant pattern.

### CD4bs bNAbs.

Fig. S2E and F shows that CD4bs-targeting antibodies VRC01 and VRC07-LS, had the most consistently potent intraparticipant neutralization profiles longitudinally. For VRC07-LS in all participants, all longitudinal IC_50_ values were less than 1 μg/mL. Interestingly, for participants 208 and 079, variants appeared to be evolving to become more sensitive to VRC01. Also, noteworthy was the subtle increase in resistance to VRC01 and VRC07-LS observed for participants 036, 186 and 318. For 3BNC117 (Fig. S1G), variants from 079 and 093 were all resistant; however, a relative loss of resistance to 3BNC117 was registered for later time point sampled variants from 079. Moreover, variants from 036, 186, and 318, appeared to be acquiring resistance to 3BNC117 over time. In another analysis, the heatmap in Fig. 5C shows that all participants studied, cross-sectionally or longitudinally, had largely conserved CD4bs signature mutation profiles. However, surprisingly, the intraparticipant neutralization profiles against the CD4bs antibodies were variable. For instance, within participant 186, the virus (186_AT_SGA5) isolated approximately 1 year post ART initiation is notably resistant to 3BNC117 but has the same signature mutation profile compared to the transmitted founder virus (186_TF_SGA9) as well as the virus that was circulating in plasma at chronic infection before treatment (186_BT_SGA1). The D99Y mutation in 318_AT_SGA1 appeared to lead to reduced sensitivity against the studied CD4bs antibodies. The S282P mutation in 102_BT_SGA6 was associated with resistance to VRC01 but had no effect on other tested CD4bs antibodies. Moreover, for participant 079, the T371K mutation was associated with increased sensitivity to 3BNC117. Surprisingly, participant 036 had similar signature mutations but the TF was more sensitive to neutralization sensitivity by CD4bs antibodies than the virus that was isolated from plasma prior to ART initiation at chronic infection phase. Unexpectedly, all the studied variants from participant 451 had the same signature mutations but varied in neutralization sensitivity against CD4bs antibodies. Interestingly, despite signature mutation profiles being associated with reduced sensitivity to CD4bs antibodies, there was simultaneous association of reduced sensitivity to CD4bs antibodies with elevated S/T counts and PNGSs, as well as domain lengths of both V1 and V4, especially for participants186 and 318. Overall, these data suggest that the intraparticipant neutralization sensitivity against CD4bs antibodies can fluctuate over time.

### MPER bNAb.

Fig. S2H shows that MPER-targeting antibody 10E8, had varied intraparticipant longitudinal neutralization profiles. Notably, the TF variants from participants 036, 318, and 451 were more resistant to 10E8 than their corresponding later-sampled variants. In contrast, for participant 186, the TF was more sensitive to 10E8 than later-sampled variants. In addition, Fig. 5D shows that there was no significant intraparticipant longitudinal variation among the studied participants. Moreover, we did not observe rare mutations associated with complete 10E8 resistance: N671T, W672L, F673L, and W680G (44). Furthermore, although the K683R signature is also associated with resistance to MPER-targeting antibodies (45, 46), it did not appear to cause resistance in this study (PID 079). Interestingly, we noted unusual V1 and V4 characteristics of variants that displayed markedly low sensitivity to 10E8 in PIDs: 036, 079, 093, 102, 186, 318, and 451 — all of whom had high ST counts and/or relatively higher PNGSs and/or relatively longer lengths.

### gp120/gp41 bNAb.

Fig. S2I shows that gp120/gp41-targeting antibody PGT151, had varied intraparticipant longitudinal neutralization profiles. Particularly, the TF variants from participants 036, 102, 186, and 318 were more sensitive to PGT151 than their variant counterparts from later time points. In contrast, the TF variant from participant 451 was more resistant to PGT151 than corresponding variants sampled later. The rest of the participants appeared to have a relatively conserved intraparticipant neutralization sensitivity over time. Moreover, as shown in the heatmap (Fig. 5E), we observed that the glycans N262, N448, N611, N616, and N637 were largely conserved longitudinally among all participants. However, we observed that in participants 186 and 651, the N448S mutation did not compromise sensitivity to PGT151. Nonetheless, in participant 318 and 186, both the N637S/K and N300G/S mutations were associated with resistance to PGT151. Contrary to the trends observed for bNAbs targeting earlier described epitopes, the unusual V1 and V4 characteristics (high ST counts and/or relatively higher PNGSs and/or relatively longer lengths) of select variants did not associate with low sensitivity to the gp120/gp41 bNAb (PGT151).

## DISCUSSION

Our phylogenetic analysis yielded results consistent with the consensus in the field that there is a stringent genetic bottleneck governing transmission (47) and that early ART reduces intrahost viral diversification over time (48). Delaying ART initiation until the chronic stage of infection allows for intrahost viral diversification (49). The current study is also consistent with an earlier study (50) suggesting that, generally, archived peripheral blood reservoir variants following suppressive ART resemble variants circulating in the peripheral blood prior to ART initiation. We also found that Env features that may impact neutralization bNAb sensitivity, including length, PNGSs and total S/T counts can be dynamic for V1 and V4 domains but relatively conserved longitudinally for V2, V3, and V5 domains. Furthermore, in some instances, the intraparticipant viral neutralization profiles were associated with changes in domain length, PNGSs, and total S/T counts, particularly for V1 and V4. These findings imply that V1 and V4 domains may be useful in viral immune evasion.

Overall, consistent with what other studies have reported (12, 51–54), the phenotypic neutralization data showed that bNAbs have heterogeneity toward neutralization of circulating HIV-1 subtype C viruses. The most effective bNAbs against the Env clones tested, with more than 80% breadth at 1 μg/mL concentration were VRC07-LS and N6.LS (both with 100% breadth) for CD4bs-targeting antibodies; PGT121 and 10-1074 (both with 80% breadth) for V3-targeting antibodies; and PGT151 (81% breadth) for the gp12/gp41 interface. The V1/V2-targeting CAP256.VRC26 and PGDM1400 had 57% and 52% breadth, respectively; while the MPER-targeting antibody 10E8 (68% breadth) and the FP-targeting N123-VRC34.01 (38% breadth) were moderately to weakly neutralizing. Whereas the use of the IC_50_ threshold of ≤1 μg/mL can guide *in vitro* neutralization breadth assessments, other studies have suggested that a combination of 3BNC117 and 10-1074 is sufficient to maintain viral suppression in sensitive individuals when the concentration of both antibodies remains ≥10 μg/mL in serum (21, 55). Other studies with a more stringent threshold toward prevention efficacy (PE) of VRC01, such as the HVTN 703/HPTN 081 (703/081), which enrolled heterosexual women in Botswana, Kenya, Malawi, Mozambique, South Africa, Tanzania, and Zimbabwe, suggested a PE of 78.6% (95% CI 17.3 to 94.4%) against viruses with IC_80_ <1 μg/mL, but offering a near zero PE for viruses with IC_80_ >1 μg/mL (56). Furthermore, based on the VRC01 AMP study data, as well as steady-state serum concentration simulations of PGT121.414.LS, PGDM1400LS, and VRC07-523LS, a recent study suggested an average PT_80_ of 200 (implying a bNAb concentration 200-fold greater than that required to prevent infection by 80% *in vitro*) as required for 90% preventative efficacy against virus acquisition (56). Although the majority of the bNAbs on the current study’s panel have not been studied in humans, our study further highlights the need to identify or engineer more bNAbs with high breadth and potency for potential clinical use.

Additionally, we found differences in neutralization sensitivity across transmitted, plasma-circulating, and reservoir variants against some bNAb classes. These data suggest that bNAbs may not be uniformly effective when applied in prevention versus treatment strategies. For example, VRC01 and PGDM1400 appear to have different patterns, whereby TF variants may be more resistant to VRC01, but more sensitive to PGDM1400 and vice-versa for variants derived for later time points. Moreover, evolution of antibody neutralization sensitivity can occur within an individual over time (57, 58) and can be compartment specific (59). In our study, instances of neutralization compartmentalization for bNAbs such as 10E8 and PGT151 were observed (such as in Fig. 4J and N, respectively). Compartmentalization was more common in individuals who commenced ART in chronic HIV-1 infection. For instance, LN compartment-derived variants were generally more sensitive than variants derived from the PBMC compartment at matching time points, notably, variants from PID 079 versus VRC01 or 10E8, and variants from PID 093 versus PGT151. However, these observations will require further confirmation in larger studies.

Moreover, we identified genetic characteristics within the Env that might predict sensitivity to bNAb (mainly within V1 and V4), suggesting that the function of these variable domains in concealing HIV-1-neutralizing epitopes is currently being underestimated (60, 61). Notably, a recent study reported an HIV-1 isolate (from an elite controller) that displayed an unusually long V1 domain in Env gp120. Upon careful analysis of this V1 domain, its stretches of S and T had positive O-glycosylation prediction (62). A follow-up cross-sectional study also suggested that the O-glycans within the V1 domain, shields HIV-1 against V3-glycan bNAbs (40). Consistent with these prior studies, our current study revealed that; among variants in PID 079, a combination of longer V1/V4 and higher S/T counts within V1/V4 were associated with reduced sensitivity to V3 or V1/V2-targeting antibodies. In addition, among variants in PID 318, longer V1 domains and higher PNGSs or S/T counts within V1 were associated with reduced sensitivity to V3-, V1/V2-, and CD4bs-targeting antibodies. Variants from PID 186 also demonstrated that V1 domains that have higher PNGSs or higher S/T counts and V4 domains that have higher S/T counts are associated with reduced sensitivity to the MPER targeting antibody 10E8. These examples, suggest that bNAb sensitivity and resistance can be mediated by very subtle Env genetic changes that are not easily identifiable, particularly when using a small sample size such as ours.

There are some limitations to our study that call for caution in the interpretation of the data. First, it would have benefited from structural biology studies. For example, to comprehensively investigate the role of structural features of Env variable domains in mediating differential shielding effects against bNAbs, future studies should employ X-ray crystallography or cryo-EM (63–66) on a representative series of gp160s generated in wild-type HEK293T cells. A comparison of these crystal structures alone and in complex with individual antibodies will help us define the adhesion pivots for these antibodies in the context of variable domain features (67). Second, because of the small sample size, this study only provided subtle hints on molecular events that could be leading to neutralization sensitivity heterogeneity; Therefore, future studies should employ a higher degree of sampling, artificial intelligence, and/or machine learning (68–70) on longitudinal sequencing data to monitor both the conserved (C1 to C5) and variable domains (V1 to V5) of the gp120 subunits. These future studies might reveal conformational dynamics of the neutralizing epitopes and help model future vaccines by exposing energetically stable or possible conformations that interact with some of the key bNAbs, including changes in glycan hole (71, 72) over time. Moreover, future studies should also seek to evaluate how V1 and/or V4 variations correlate with autologous nAb response specificity. Finally, this study only evaluated the dynamics within HIV-1 subtype C Envs; therefore, future studies should also evaluate these dynamics within individuals infected with other HIV-1 subtypes. Proof-of-concept studies have demonstrated that passive administration of bNAb(s) can prevent HIV-1 acquisition or suppress viremia in humans when the targeted viruses are susceptible to the bNAbs (19, 73–75). Moreover, promising studies with various bNAbs are ongoing (NCT05281510, NCT04983030, NCT04871113, NCT05079451). Given the additional neutralization sensitivity determinants and ongoing evolution of Env within an individual suggested by this study, multispecific antibodies or bNAb combinations will most likely be required to produce the necessary breadth for effective protection.

In conclusion, our results suggest that VRC07-LS, N6.LS, VRC01, PGT151, 10-1074, and PGT121 are potent antibodies that should be recommended for future randomized AMP and treatment studies targeting high-prevalence HIV-1 subtype C regions like South Africa. This study also provides evidence of unique neutralization phenotypes between TF and chronic or latent reservoir Env variants against current bNAbs. Taken together, these findings support the need for continuous intrahost surveillance of evolving viruses during subtype C infections, to identify bNAb combinations that will optimally traverse and overcome the evolving viral genetic diversity.

## MATERIALS AND METHODS

### Study participants.

Biological specimens were collected from nine individuals under the Females Rising through Education, Support, and Health (FRESH) hyperacute HIV-1 subtype C infection study cohort based in Durban, South Africa. Participants in the FRESH cohort were HIV uninfected 18 to 23-year-old women who attended a twice-weekly socioeconomic empowerment program (76, 77). Each appointment (twice per week) included a finger-prick blood draw sample for HIV RNA testing, with women identified as HIV-1 RNA positive entering into an acute phase with more intensive sampling. Biological sampling of HIV-positive individuals was maintained throughout the acute and chronic stages (77). Six of the participants (PID 036; 093; 102; 186, 208; and 318) began ART at the chronic stage of infection. Two participants (PID 451 and 651) initiated ART within 1 week of first detection of HIV-1 positive plasma viremia following changes in the South African national treatment study guidelines. The remaining participant (PID 079) was ART-naive throughout the duration of the study. HIV-1 variants were sampled from plasma prior to ART initiation. For those participants who initiated treatment at the chronic stage, as well as those who initiated ART following HIV-1 detection, variants were sampled in PBMCs at approximately 1-year post ART initiation (AT). In addition, variants from the LN tissue samples collected from three participants (PID 079 at 1,296 days postinfection; PID 093 at 1,044 days postinfection; and PID 651 at 484 days postinfection) were analyzed. This allowed us to longitudinally assess HIV-1 *env* variants immediately (within 1 week) postinfection (termed TF virus for all nine participants); at approximately 300 to 1,709 days postinfection but prior to initiating ART (median = 724 days) for seven participants; and approximately 1 year post ART initiation for eight participants. The study was approved by the Biomedical Research Ethics Committee (BREC) of the University of KwaZulu-Natal (reference no: BE278/18) and participants provided written informed consent. The virological and immunological characteristics of study participants are shown in Table 2.

**TABLE 2:** Virological and immunological characteristics of study participants alongside sequences and clones generated

PID	Treatment start day^*a*^	DPOPV	Plasma viral load (copies/mL)	CD4 count (cells/μL)	Sampled compartment	Total SGA sequences	Representative *env* clones^*b*^
036	724	3 665 1,102	2,400,000 22,000 <20	204 213 352	Plasma Plasma PBMC	18 6 9	1 3 3
079	1,709	1 1,318 1,318 1,586	240,000 22,000 22,000 37,000	637 355 355 198	Plasma Lymph Node PBMC Plasma	16 15 14 11	1 3 5 3
093	310	1 252 693 1,375 1,375	31,000 41,000 <20 <20 <20	493 216 492 464 464	Plasma Plasma PBMC Lymph Node PBMC	34 12 4 13 5	1 3 2 3 3
102	809	3 711 1,095	6,800,000 100,000 <20	814 589 598	Plasma Plasma PBMC	17 9 4	- 3 3
186	347	4 333 745	6,300,000 210,000 110,000	306 287 208	Plasma Plasma PBMC	21 10 9	1 2 3
208	427	1 413 868	46,000 82,000 <20	390 327 471	Plasma Plasma PBMC	19 7 7	1 3 2
318	1,059	3 990 1,417	160,000 7,900 <20	911 700 663	Plasma Plasma PBMC	25 5 5	1 3 3
451	1	1 328	62,000 <20	434 738	Plasma PBMC	2 14	1 3
651	1	1 338 484	3,900 <20 <20	851 545 623	Plasma PBMC Lymph node^*c*^	4 8 3	4 3

a Day of ART initiation measured from post detection of plasma viremia.

b Based on phylogenetic clustering, a median of three representative clones for subsequent neutralization testing were generated per time point.

*^c^*Limited sample; bulk PCR product cloned. DPOPV, days post first detection of plasma viremia; SGA, single genome amplicon.

### Nucleic acid extraction and cDNA synthesis.

Integrated proviral HIV-1 DNA was extracted using the Qiagen DNeasy blood and tissue kit (Qiagen, Hilden, Germany) from peripheral blood mononuclear cells of clinical samples. The DNA was eluted from the spin column membrane using 200 μL of AE buffer and stored at −20°C for use in the next step. The MasterPure complete DNA and RNA purification kit (Lucigen, Middleton, WI, USA) was used to extract total DNA from the lymph node samples. HIV-1 mRNA was also extracted from plasma using the QIAamp Viral RNA minikit (Qiagen, Hilden, Germany), and cDNA was prepared by reverse transcription-PCR (RT-PCR) using a Superscript IV First-Strand synthesis kit according to the manufacturer’s protocol (Thermo Fisher Scientific, Waltham, MA, USA) and the gene-specific primer OFM19 (5′-GCA CTC AAG GCA AGC TTT ATT GAG GCT TA-3′). Reverse transcription was carried out for 1.5 h at 50°C, and cDNA products were stored at −20°C. The resultant DNA or cDNA was serially diluted to generate single genomes of the HIV-1 *env* gene through nested PCR.

### Single genome amplification and cloning of HIV-1 *env*.

We performed a nested single-genome amplification (SGA) of the HIV-1 *env* gene using an endpoint dilution series whereby by Poisson distribution, the dilution resulting in no more than 30% of positive PCRs was considered to contain only a single genome of HIV-1 (78). The diluted template was used in the first-round of the nested single-genome amplification using the following primers: OFM19, 5′-GCA CTC AAG GCA AGC TTT ATT GAG GCT TA-3′ and VIF1, 5′- GGG TTT ATT ACA GGG ACA GCA GA-3′ (79). Sample DNA was used as the template in a 20 μL reaction volume containing 10× buffer, 2 mM MgSO_4_, 10 mM dNTPs, 10 pmol/μL of each primer, 5 U/μL Platinum HF DNA polymerase (Thermo Fisher, Waltham MA, USA), and nuclease-free H_2_O. PCR was carried out using the following program: 1 cycle of 94°C for 4 min; 35 cycles of 94°C for 15 s, 55°C for 30 s, and 68°C for 4 min; a final extension of 68°C for 20 min; then held at 4°C.

The second-round of the nested SGA was done using the in-house modified primers: Env1A(3rd)GA, 5′-CTC CGA TCC AGT ACC CTT CAC CGG CTT AGG CAT CTC CTA T-3′ and Env1M(3rd)GA, 5′-GCA GAA TTG TCT TGA CCC TTT AGC CCT TCC AGT CCC CCC-3′. The primers were designed to incorporate a 15-bp overlap (shown in in the underlined segments) which were complementary to the ends of the linearized pcDNA 3.1/V5-His-TOPO sequence using the Gibson-Assembly approach (80). The first-round PCR product was used as the template in a 25-μL reaction volume containing 5X Phusion buffer, 2 mM MgSO_4_, 10 mM dNTPs, 20 pmol/μL of each primer, 5 U/μL Phusion HF *Taq* polymerase (Thermo Fisher, Waltham MA, USA), and nuclease-free H_2_O. PCR was carried out using: 1 cycle of 98°C for 30 s; 35 cycles of 98°C for 10 s, 65°C for 30 s, and 72°C for 4 min; a final extension of 72°C for 10 min; then held at 4°C. The amplimers were visualized on a 2% agarose gel (Invitrogen, Carlsbad, CA, USA) in 1× Tris-borate-EDTA (TBE) buffer (Invitrogen, Grand Island, NY, USA) containing 0.5× GelRed (Biotium, Fremont, CA, USA) and run at 120 V for 1 h. The resulting PCR amplicons were excised and purified using the Qiagen QIAquick gel extraction kit (Thermo Fisher Scientific, Inc., USA) and ligated into the pcDNA 3.1/V5-His-TOPO using the NEBuilder HiFi DNA assembly master mix (New England BioLabs, Ipswich, MA, USA).

### Sequence and phylogenetic analysis.

DNA sequences were edited using Sequencher version 5.4.6 (Gene Codes Corporation, Ann Arbor, MI, USA) and further analyzed using Geneious Prime software (Biomatters, Auckland, New Zealand). Variable loop lengths and the total serine/threonine (S/T) counts were determined within each individual variable region (V1 to V5). The variable region coordinates were determined using the following HXB2 amino acid (AA) positions (GenBank accession number K03455): V1, AA 127 to 156; V2, AA 160 to 195; V3, AA 300 to 328; V4, AA 393 to 414; and V5, AA 460 to 470 (81). The number of potential N-linked glycosylation sites (PNGSs) were ascertained using the N-Glycosite tool from the Los Alamos HIV database website (http://www.hiv.lanl.gov/content/sequence/GLYCOSITE/glycosite.html; accessed 10 April 2020). The variable loop length and the total count of S/T residues within the V1, V2, V3, V4, and V5 loops were compared between different clusters and time points for each sequence. Sequence logo plots were drawn in WebLogo (http://weblogo.berkeley.edu/logo.cgi; accessed 2 May 2021). The intra- and interparticipant evolutionary analyses were conducted in MEGA X (82) using the maximum likelihood method and Jones-Taylor-Thornton (JTT) matrix-based model (83).

### Envelope-pseudotyped virus production.

Pseudotyped viruses were prepared by the cotransfection of an envelope-expressing plasmid with an *env*-deleted HIV-1 backbone plasmid (pSG3ΔEnv) into 293T cells in T75 culture flasks using a FuGENE6 transfection kit (Promega Inc., Madison, WI, USA). Cell supernatants containing pseudotyped viruses were harvested 24 h posttransfection and then stored at 80°C until further use. TCID_50_ was assessed through titration of the pseudovirus stocks in quadruplicate 5-fold serial dilutions in a 96-well plate. TZM-bl cells containing dextran-hydrochloride (DEAE) were added to the virus, and TZM-bl cells not treated with the virus were used as a negative control. The plate was incubated at 37°C, 95% relative humidity, and 5% CO_2_ for 48 h. After incubation, growth media were removed and replaced with the Bright-Glo luciferase assay substrate (Promega Inc., Madison, WI, USA) and viral transfectivity was measured as relative light units (RLU) using a GloMax Navigator luminometer (Promega Inc., Madison, WI, USA). The neutralization assays employed an infectious dosage that produced between 50,000 and 150,000 RLU. We chose a dosage that provided at least 10 times the baseline RLU for pseudoviruses that did not exceed 50,000 to 150,000 RLU (84).

### TZM-bl HIV neutralization antibody assay.

Neutralization was measured as a reduction in luciferase gene expression after a single-round infection of TZM-bl cells (NIH AIDS Reagent Program) with Env-pseudotyped viruses (85). A 5-fold dilution series was used for each antibody and the maximum antibody concentration tested for each antibody was 25 μg/mL. Luciferase activity was measured after incubation at 37°C, 95% relative humidity, and 5% CO_2_ for 48 h. Titers were calculated as the inhibitory concentration (IC_50_) causing 50% reduction of RLU with respect to the virus control wells (untreated virus). Reported neutralization titers are the average of two or more titers for any given virus-antibody combination.

### Statistical analysis.

Statistical analysis was performed using GraphPad Prism version 9 software (San Diego, CA, USA). Statistical comparisons between sequence characteristics and neutralization sensitivities were performed using the Mann-Whitney U test and unpaired *t* test, where *P* < 0.05 was considered significant.

### Data availability.

All sequences analyzed in this study have been deposited in GenBank under accession numbers OP121239 to OP121555.

## References

[1] Liu Y, Cao W, Sun M, Li T. 2020. Broadly neutralizing antibodies for HIV-1: efficacies, challenges and opportunities. Emerg Microbes Infect 9:194–206. 10.1080/22221751.2020.1713707. 31985356 PMC7040474

[2] Julg B, Barouch D. 2021. Broadly neutralizing antibodies for HIV-1 prevention and therapy. Semin Immunol 51:101475. and Elsevier. 10.1016/j.smim.2021.101475. 33858765

[3] Parker Miller E, Finkelstein MT, Erdman MC, Seth PC, Fera D. 2021. A structural update of neutralizing epitopes on the HIV envelope, a moving target. Viruses 13:1774. 10.3390/v13091774. 34578355 PMC8472920

[4] McFarland EJ, Cunningham CK, Muresan P, Capparelli EV, Perlowski C, Morgan P, Smith B, Hazra R, Purdue L, Harding PA, Theron G, Mujuru H, Agwu A, Purswani M, Rathore MH, Flach B, Taylor A, Lin BC, McDermott AB, Mascola JR, Graham BS, Rossouw M, Rossouw L, Louw J, Vhembo T, Mhembere TP, Matibe P, Mahmoudi S, Maldonado A, Maraqa N, Baig MM, Rogo T, Cavallo M, Collinson-Streng A, Anderson T, Golden WC, Persaud D, Puga AM, Robinson L-G, Eysallenne Z, Leon D, Paul ME, McMullen-Jackson C, Buschur S, Pontifes M, Sung J, Glenny C, Dunn J, Navarro K, International Maternal Pediatric Adolescent AIDS Clinical Trials Network (IMPAACT) P1112 Team. 2021. Safety, tolerability, and pharmacokinetics of a long-acting broadly neutralizing HIV-1 monoclonal antibody VRC01LS in HIV-1-exposed newborn infants. The J Infectious Diseases 224:1916–1924. 10.1093/infdis/jiab229. 34009371 PMC8643399

[5] Spencer DA, Shapiro MB, Haigwood NL, Hessell AJ. 2021. Advancing HIV broadly neutralizing antibodies: from discovery to the clinic. Front Public Health 9:610. 10.3389/fpubh.2021.690017. PMC8187619 34123998

[6] Schoofs T, Klein F, Braunschweig M, Kreider EF, Feldmann A, Nogueira L, Oliveira T, Lorenzi JCC, Parrish EH, Learn GH, West AP, Bjorkman PJ, Schlesinger SJ, Seaman MS, Czartoski J, McElrath MJ, Pfeifer N, Hahn BH, Caskey M, Nussenzweig MC. 2016. HIV-1 therapy with monoclonal antibody 3BNC117 elicits host immune responses against HIV-1. Science 352:997–1001. 10.1126/science.aaf0972. 27199429 PMC5151174

[7] Nishimura Y, Gautam R, Chun T-W, Sadjadpour R, Foulds KE, Shingai M, Klein F, Gazumyan A, Golijanin J, Donaldson M, Donau OK, Plishka RJ, Buckler-White A, Seaman MS, Lifson JD, Koup RA, Fauci AS, Nussenzweig MC, Martin MA. 2017. Early antibody therapy can induce long-lasting immunity to SHIV. Nature 543:559–563. 10.1038/nature21435. 28289286 PMC5458531

[8] Sliepen K, Medina-Ramírez M, Yasmeen A, Moore JP, Klasse PJ, Sanders RW. 2015. Binding of inferred germline precursors of broadly neutralizing HIV-1 antibodies to native-like envelope trimers. Virology 486:116–120. 10.1016/j.virol.2015.08.002. 26433050 PMC4712445

[9] Ward AB, Wilson IA. 2015. Insights into the trimeric HIV-1 envelope glycoprotein structure. Trends Biochem Sci 40:101–107. 10.1016/j.tibs.2014.12.006. 25600289 PMC4310573

[10] Haynes BF, Burton DR, Mascola JR. 2019. Multiple roles for HIV broadly neutralizing antibodies. Sci Transl Med 11. 10.1126/scitranslmed.aaz2686. PMC7171597 31666399

[11] Wibmer CK, Gorman J, Ozorowski G, Bhiman JN, Sheward DJ, Elliott DH, Rouelle J, Smira A, Joyce MG, Ndabambi N, Druz A, Asokan M, Burton DR, Connors M, Abdool Karim SS, Mascola JR, Robinson JE, Ward AB, Williamson C, Kwong PD, Morris L, Moore PL. 2017. Structure and recognition of a novel HIV-1 gp120-gp41 interface antibody that caused MPER exposure through viral escape. PLoS Pathog 13:e1006074. 10.1371/journal.ppat.1006074. 28076415 PMC5226681

[12] Bonsignori M, Liao H-X, Gao F, Williams WB, Alam SM, Montefiori DC, Haynes BF. 2017. Antibody-virus co-evolution in HIV infection: paths for HIV vaccine development. Immunol Rev 275:145–160. 10.1111/imr.12509. 28133802 PMC5302796

[13] Kong R, Xu K, Zhou T, Acharya P, Lemmin T, Liu K, Ozorowski G, Soto C, Taft JD, Bailer RT, Cale EM, Chen L, Choi CW, Chuang G-Y, Doria-Rose NA, Druz A, Georgiev IS, Gorman J, Huang J, Joyce MG, Louder MK, Ma X, McKee K, O'Dell S, Pancera M, Yang Y, Blanchard SC, Mothes W, Burton DR, Koff WC, Connors M, Ward AB, Kwong PD, Mascola JR. 2016. Fusion peptide of HIV-1 as a site of vulnerability to neutralizing antibody. Science 352:828–833. 10.1126/science.aae0474. 27174988 PMC4917739

[14] Stephenson KE, Wagh K, Korber B, Barouch DH. 2020. Vaccines and broadly neutralizing antibodies for HIV-1 prevention. Annu Rev Immunol 38:673–703. 10.1146/annurev-immunol-080219-023629. 32340576 PMC7375352

[15] González A, Güémez A. Use of broadly neutralizing antibodies as an alternative treatment for HIV-1, what the clinical trials show: a review. BioTecnología 25.

[16] Xu L, Pegu A, Rao E, Doria-Rose N, Beninga J, McKee K, Lord DM, Wei RR, Deng G, Louder M, Schmidt SD, Mankoff Z, Wu L, Asokan M, Beil C, Lange C, Leuschner WD, Kruip J, Sendak R, Kwon YD, Zhou T, Chen X, Bailer RT, Wang K, Choe M, Tartaglia LJ, Barouch DH, O'Dell S, Todd J-P, Burton DR, Roederer M, Connors M, Koup RA, Kwong PD, Yang Z-Y, Mascola JR, Nabel GJ. 2017. Trispecific broadly neutralizing HIV antibodies mediate potent SHIV protection in macaques. Science 358:85–90. 10.1126/science.aan8630. 28931639 PMC5978417

[17] Julg B, Liu P-T, Wagh K, Fischer WM, Abbink P, Mercado NB, Whitney JB, Nkolola JP, McMahan K, Tartaglia LJ, Borducchi EN, Khatiwada S, Kamath M, LeSuer JA, Seaman MS, Schmidt SD, Mascola JR, Burton DR, Korber BT, Barouch DH. 2017. Protection against a mixed SHIV challenge by a broadly neutralizing antibody cocktail. Sci Transl Med 9. 10.1126/scitranslmed.aao4235. PMC5747528 28931655

[18] Julg B, Tartaglia LJ, Keele BF, Wagh K, Pegu A, Sok D, Abbink P, Schmidt SD, Wang K, Chen X, Joyce MG, Georgiev IS, Choe M, Kwong PD, Doria-Rose NA, Le K, Louder MK, Bailer RT, Moore PL, Korber B, Seaman MS, Abdool Karim SS, Morris L, Koup RA, Mascola JR, Burton DR, Barouch DH. 2017. Broadly neutralizing antibodies targeting the HIV-1 envelope V2 apex confer protection against a clade C SHIV challenge. Sci Transl Med 9. 10.1126/scitranslmed.aal1321. PMC5755978 28878010

[19] Corey L, Gilbert PB, Juraska M, Montefiori DC, Morris L, Karuna ST, Edupuganti S, Mgodi NM, deCamp AC, Rudnicki E, Huang Y, Gonzales P, Cabello R, Orrell C, Lama JR, Laher F, Lazarus EM, Sanchez J, Frank I, Hinojosa J, Sobieszczyk ME, Marshall KE, Mukwekwerere PG, Makhema J, Baden LR, Mullins JI, Williamson C, Hural J, McElrath MJ, Bentley C, Takuva S, Gomez Lorenzo MM, Burns DN, Espy N, Randhawa AK, Kochar N, Piwowar-Manning E, Donnell DJ, Sista N, Andrew P, Kublin JG, Gray G, Ledgerwood JE, Mascola JR, Cohen MS, HVTN 704/HPTN 085 and HVTN 703/HPTN 081 Study Teams. 2021. Two randomized trials of neutralizing antibodies to prevent HIV-1 acquisition. N Engl J Med 384:1003–1014. 10.1056/NEJMoa2031738. 33730454 PMC8189692

[20] Caskey M, Klein F, Nussenzweig MC. 2019. Broadly neutralizing anti-HIV-1 monoclonal antibodies in the clinic. Nat Med 25:547–553. 10.1038/s41591-019-0412-8. 30936546 PMC7322694

[21] Bar-On Y, Gruell H, Schoofs T, Pai JA, Nogueira L, Butler AL, Millard K, Lehmann C, Suárez I, Oliveira TY, Karagounis T, Cohen YZ, Wyen C, Scholten S, Handl L, Belblidia S, Dizon JP, Vehreschild JJ, Witmer-Pack M, Shimeliovich I, Jain K, Fiddike K, Seaton KE, Yates NL, Horowitz J, Gulick RM, Pfeifer N, Tomaras GD, Seaman MS, Fätkenheuer G, Caskey M, Klein F, Nussenzweig MC. 2018. Safety and antiviral activity of combination HIV-1 broadly neutralizing antibodies in viremic individuals. Nat Med 24:1701–1707. 10.1038/s41591-018-0186-4. 30258217 PMC6221973

[22] Chen Y, Shen Z, Feng Y, Ruan Y, Li J, Tang S, Tang K, Liang S, Pang X, McNeil EB, Xing H, Chongsuvivatwong V, Lin M, Lan G. 2021. HIV-1 subtype diversity and transmission strain source among men who have sex with men in Guangxi, China. Sci Rep 11:1–8. 10.1038/s41598-021-87745-3. 33859273 PMC8050077

[23] Wilson A, Lynch RM. 2020. Embracing diversity: how can broadly neutralizing antibodies effectively target a diverse HIV-1 reservoir? Current Opinion in Pharmacology 54:173–178. 10.1016/j.coph.2020.10.004. 33189993

[24] De Scheerder M-A, Vrancken B, Dellicour S, Schlub T, Lee E, Shao W, Rutsaert S, Verhofstede C, Kerre T, Malfait T, Hemelsoet D, Coppens M, Dhondt A, De Looze D, Vermassen F, Lemey P, Palmer S, Vandekerckhove L. 2019. HIV rebound is predominantly fueled by genetically identical viral expansions from diverse reservoirs. Cell Host Microbe 26:347–358.e7. 10.1016/j.chom.2019.08.003. 31471273 PMC11021134

[25] Rife Magalis B, Nolan DJ, Autissier P, Burdo TH, Williams KC, Salemi M. 2017. Insights into the impact of CD8+ immune modulation on human immunodeficiency virus evolutionary dynamics in distinct anatomical compartments by using simian immunodeficiency virus-infected macaque models of AIDS progression. J Virol 91:e01162-17. 10.1128/JVI.01162-17. 28931681 PMC5686727

[26] Alves BM, Siqueira JD, Prellwitz IM, Botelho OM, Da Hora VP, Sanabani S, Recordon-Pinson P, Fleury H, Soares EA, Soares MA. 2019. Estimating HIV-1 genetic diversity in Brazil through next-generation sequencing. Front Microbiol 10:749. 10.3389/fmicb.2019.00749. 31024510 PMC6465556

[27] Brown RJP, Peters PJ, Caron C, Gonzalez-Perez MP, Stones L, Ankghuambom C, Pondei K, McClure CP, Alemnji G, Taylor S, Sharp PM, Clapham PR, Ball JK. 2011. Intercompartmental recombination of HIV-1 contributes to env intrahost diversity and modulates viral tropism and sensitivity to entry inhibitors. J Virol 85:6024–6037. 10.1128/JVI.00131-11. 21471230 PMC3126287

[28] Boutwell CL, Rolland MM, Herbeck JT, Mullins JI, Allen TM. 2010. Viral evolution and escape during acute HIV-1 infection. J Infect Dis 202:S309–S314. 10.1086/655653. 20846038 PMC2945609

[29] Barton JP, Goonetilleke N, Butler TC, Walker BD, McMichael AJ, Chakraborty AK. 2016. Relative rate and location of intra-host HIV evolution to evade cellular immunity are predictable. Nat Commun 7:11660–11610. 10.1038/ncomms11660. 27212475 PMC4879252

[30] Hu Y, Zou S, Wang Z, Liu Y, Ren L, Hao Y, Sun S, Hu X, Ruan Y, Ma L, Shao Y, Hong K. 2021. Virus evolution and neutralization sensitivity in an HIV-1 subtype B′ infected plasma donor with broadly neutralizing activity. Vaccines 9:311. 10.3390/vaccines9040311. 33805985 PMC8064334

[31] Rossignol ED, Dugast A-S, Compere H, Cottrell CA, Copps J, Lin S, Cizmeci D, Seaman MS, Ackerman ME, Ward AB, Alter G, Julg B. 2021. Mining HIV controllers for broad and functional antibodies to recognize and eliminate HIV-infected cells. Cell Rep 35:109167. 10.1016/j.celrep.2021.109167. 34038720 PMC8196545

[32] Rajashekar JK, Richard J, Beloor J, Prévost J, Anand SP, Beaudoin-Bussières G, Shan L, Herndler-Brandstetter D, Gendron-Lepage G, Medjahed H, Bourassa C, Gaudette F, Ullah I, Symmes K, Peric A, Lindemuth E, Bibollet-Ruche F, Park J, Chen H-C, Kaufmann DE, Hahn BH, Sodroski J, Pazgier M, Flavell RA, Smith AB, Finzi A, Kumar P. 2021. Modulating HIV-1 envelope glycoprotein conformation to decrease the HIV-1 reservoir. Cell Host Microbe 29:904–916.e6. 10.1016/j.chom.2021.04.014. 34019804 PMC8214472

[33] Zhang P, Kwon AL, Guzzo C, Liu Q, Schmeisser H, Miao H, Lin Y, Cimbro R, Huang J, Connors M, Schmidt SD, Dolan MA, Armstrong AA, Lusso P. 2021. Functional anatomy of the trimer apex reveals key hydrophobic constraints that maintain the HIV-1 envelope spike in a closed state. mBio 12. 10.1128/mBio.00090-21. PMC8092198 33785631

[34] Li Y, Guo Y, Cheng H, Zeng X, Zhang X, Sang P, Chen B, Yang L. 2022. Deciphering gp120 sequence variation and structural dynamics in HIV neutralization phenotype by molecular dynamics simulations and graph machine learning. Proteins 90:1413–1424. 10.1002/prot.26322. 35171521

[35] Sahoo A, Hodge EA, LaBranche CC, Styles TM, Shen X, Cheedarla N, Shiferaw A, Ozorowski G, Lee W-H, Ward AB, Tomaras GD, Montefiori DC, Irvine DJ, Lee KK, Amara RR. 2022. Structure-guided changes at the V2 apex of HIV-1 clade C trimer enhance elicitation of autologous neutralizing and broad V1V2-scaffold antibodies. Cell Rep 38:110436. 10.1016/j.celrep.2022.110436. 35235790 PMC8982139

[36] van Gils MJ, Bunnik EM, Boeser-Nunnink BD, Burger JA, Terlouw-Klein M, Verwer N, Schuitemaker H. 2011. Longer V1V2 region with increased number of potential N-linked glycosylation sites in the HIV-1 envelope glycoprotein protects against HIV-specific neutralizing antibodies. J Virol 85:6986–6995. 10.1128/JVI.00268-11. 21593147 PMC3126602

[37] Ringe R, Phogat S, Bhattacharya J. 2012. Subtle alteration of residues including N-linked glycans in V2 loop modulate HIV-1 neutralization by PG9 and PG16 monoclonal antibodies. Virology 426:34–41. 10.1016/j.virol.2012.01.011. 22314018

[38] Sutar J, Deshpande S, Mullick R, Hingankar N, Patel V, Bhattacharya J. 2021. Geospatial HIV-1 subtype C gp120 sequence diversity and its predicted impact on broadly neutralizing antibody sensitivity. PLoS One 16:e0251969. 10.1371/journal.pone.0251969. 34029329 PMC8143386

[39] Khairnar A, Sunsunwal S, Babu P, Ramya TNC. 2021. Novel serine/threonine-O-glycosylation with N-acetylneuraminic acid and 3-deoxy-D-manno-octulosonic acid by bacterial flagellin glycosyltransferases. Glycobiology 31:288–306. 10.1093/glycob/cwaa084. 32886756

[40] Silver ZA, Antonopoulos A, Haslam SM, Dell A, Dickinson GM, Seaman MS, Desrosiers RC. 2020. Discovery of O-linked carbohydrate on HIV-1 envelope and its role in shielding against one category of broadly neutralizing antibodies. Cell Rep 30:1862–1869.e4. 10.1016/j.celrep.2020.01.056. 32049016 PMC7904042

[41] Kozarsky K, Penman M, Basiripour L, Haseltine W, Sodroski J, Krieger M. 1989. Glycosylation and processing of the human immunodeficiency virus type 1 envelope protein. J Acquired Immune Deficiency Syndromes 2:163–169. 2649653

[42] Yu W-H, Su D, Torabi J, Fennessey CM, Shiakolas A, Lynch R, Chun T-W, Doria-Rose N, Alter G, Seaman MS, Keele BF, Lauffenburger DA, Julg B. 2019. Predicting the broadly neutralizing antibody susceptibility of the HIV reservoir. JCI Insight 4. 10.1172/jci.insight.130153. PMC6777915 31484826

[43] Behrens A-J, Vasiljevic S, Pritchard LK, Harvey DJ, Andev RS, Krumm SA, Struwe WB, Cupo A, Kumar A, Zitzmann N, Seabright GE, Kramer HB, Spencer DIR, Royle L, Lee JH, Klasse PJ, Burton DR, Wilson IA, Ward AB, Sanders RW, Moore JP, Doores KJ, Crispin M. 2016. Composition and antigenic effects of individual glycan sites of a trimeric HIV-1 envelope glycoprotein. Cell Rep 14:2695–2706. 10.1016/j.celrep.2016.02.058. 26972002 PMC4805854

[44] Bricault CA, Yusim K, Seaman MS, Yoon H, Theiler J, Giorgi EE, Wagh K, Theiler M, Hraber P, Macke JP, Kreider EF, Learn GH, Hahn BH, Scheid JF, Kovacs JM, Shields JL, Lavine CL, Ghantous F, Rist M, Bayne MG, Neubauer GH, McMahan K, Peng H, Chéneau C, Jones JJ, Zeng J, Ochsenbauer C, Nkolola JP, Stephenson KE, Chen B, Gnanakaran S, Bonsignori M, Williams LD, Haynes BF, Doria-Rose N, Mascola JR, Montefiori DC, Barouch DH, Korber B. 2019. HIV-1 neutralizing antibody signatures and application to epitope-targeted vaccine design. Cell Host Microbe 25:59–72.e8. 10.1016/j.chom.2018.12.001. 30629920 PMC6331341

[45] Rademeyer C, Korber B, Seaman MS, Giorgi EE, Thebus R, Robles A, Sheward DJ, Wagh K, Garrity J, Carey BR, Gao H, Greene KM, Tang H, Bandawe GP, Marais JC, Diphoko TE, Hraber P, Tumba N, Moore PL, Gray GE, Kublin J, McElrath MJ, Vermeulen M, Middelkoop K, Bekker L-G, Hoelscher M, Maboko L, Makhema J, Robb ML, Abdool Karim S, Abdool Karim Q, Kim JH, Hahn BH, Gao F, Swanstrom R, Morris L, Montefiori DC, Williamson C. 2016. Features of recently transmitted HIV-1 clade C viruses that impact antibody recognition: implications for active and passive immunization. PLoS Pathog 12:e1005742. 10.1371/journal.ppat.1005742. 27434311 PMC4951126

[46] Yin L, Chang K-F, Nakamura KJ, Kuhn L, Aldrovandi GM, Goodenow MM. 2021. Unique genotypic features of HIV-1 C gp41 membrane proximal external region variants during pregnancy relate to mother-to-child transmission via breastfeeding. J Clin Pediatr Neonatol 1:9–20. 34553192 10.46439/pediatrics.1.003 PMC8454918

[47] Beretta M, Moreau A, Bouvin-Pley M, Essat A, Goujard C, Chaix M-L, Hue S, Meyer L, Barin F, Braibant M, ANRS 06 Primo Cohort. 2018. Phenotypic properties of envelope glycoproteins of transmitted HIV-1 variants from patients belonging to transmission chains. AIDS 32:1917–1926. 10.1097/QAD.0000000000001906. 29927786

[48] Lee GQ, Reddy K, Einkauf KB, Gounder K, Chevalier JM, Dong KL, Walker BD, Yu XG, Ndung’u T, Lichterfeld M. 2019. HIV-1 DNA sequence diversity and evolution during acute subtype C infection. Nat Commun 10:1–11. 10.1038/s41467-019-10659-2. 31227699 PMC6588551

[49] Novitsky V, Wang R, Rossenkhan R, Moyo S, Essex M. 2013. Intra-host evolutionary rates in HIV-1C env and gag during primary infection. Infect Genet Evol 19:361–368. 10.1016/j.meegid.2013.02.023. 23523818 PMC3759599

[50] Abrahams M-R, Joseph SB, Garrett N, Tyers L, Moeser M, Archin N, Council OD, Matten D, Zhou S, Doolabh D, Anthony C, Goonetilleke N, Karim SA, Margolis DM, Pond SK, Williamson C, Swanstrom R. 2019. The replication-competent HIV-1 latent reservoir is primarily established near the time of therapy initiation. Sci Transl Med 11. 10.1126/scitranslmed.aaw5589. PMC7233356 31597754

[51] Li H, Zony C, Chen P, Chen BK. 2017. Reduced potency and incomplete neutralization of broadly neutralizing antibodies against cell-to-cell transmission of HIV-1 with transmitted founder Envs. J Virol 91:e02425-16. 10.1128/JVI.02425-16. 28148796 PMC5391450

[52] Bhiman JN, Anthony C, Doria-Rose NA, Karimanzira O, Schramm CA, Khoza T, Kitchin D, Botha G, Gorman J, Garrett NJ, Abdool Karim SS, Shapiro L, Williamson C, Kwong PD, Mascola JR, Morris L, Moore PL. 2015. Viral variants that initiate and drive maturation of V1V2-directed HIV-1 broadly neutralizing antibodies. Nat Med 21:1332–1336. 10.1038/nm.3963. 26457756 PMC4637988

[53] Mkhize N, Mapengo RE, Bekker V, Modise T, Kgagudi P, Lambson BE, Kaldine H, van Dorsten RT, Mgodi N, Karuna S, Edupuganti S, Corey L, Cohen MS, Hural J, McElrath J. 2021. Neutralization profiles of HIV-1 subtype C breakthrough viruses from the Southern African VRC01 AMP trial (HVTN 703/HPTN 081). J the International AIDS Society 24:9–10.

[54] Wilson A, Shakhtour L, Ward A, Ren Y, Recarey M, Stevenson E, Korom M, Kovacs C, Benko E, Jones RB, Lynch RM. 2021. Characterizing the relationship between neutralization sensitivity and env gene diversity during ART suppression. Front Immunol 12:710327. 10.3389/fimmu.2021.710327. 34603284 PMC8479156

[55] Mendoza P, Gruell H, Nogueira L, Pai JA, Butler AL, Millard K, Lehmann C, Suárez I, Oliveira TY, Lorenzi JCC, Cohen YZ, Wyen C, Kümmerle T, Karagounis T, Lu C-L, Handl L, Unson-O'Brien C, Patel R, Ruping C, Schlotz M, Witmer-Pack M, Shimeliovich I, Kremer G, Thomas E, Seaton KE, Horowitz J, West AP, Bjorkman PJ, Tomaras GD, Gulick RM, Pfeifer N, Fätkenheuer G, Seaman MS, Klein F, Caskey M, Nussenzweig MC. 2018. Combination therapy with anti-HIV-1 antibodies maintains viral suppression. Nature 561:479–484. 10.1038/s41586-018-0531-2. 30258136 PMC6166473

[56] Gilbert PB, Huang Y, deCamp AC, Karuna S, Zhang Y, Magaret CA, Giorgi EE, Korber B, Edlefsen PT, Rossenkhan R, Juraska M, Rudnicki E, Kochar N, Huang Y, Carpp LN, Barouch DH, Mkhize NN, Hermanus T, Kgagudi P, Bekker V, Kaldine H, Mapengo RE, Eaton A, Domin E, West C, Feng W, Tang H, Seaton KE, Heptinstall J, Brackett C, Chiong K, Tomaras GD, Andrew P, Mayer BT, Reeves DB, Sobieszczyk ME, Garrett N, Sanchez J, Gay C, Makhema J, Williamson C, Mullins JI, Hural J, Cohen MS, Corey L, Montefiori DC, Morris L. 2022. Neutralization titer biomarker for antibody-mediated prevention of HIV-1 acquisition. Nat Med 28:1924–1932. 10.1038/s41591-022-01953-6. 35995954 PMC9499869

[57] Rantalainen K, Berndsen ZT, Murrell S, Cao L, Omorodion O, Torres JL, Wu M, Umotoy J, Copps J, Poignard P, Landais E, Paulson JC, Wilson IA, Ward AB. 2018. Co-evolution of HIV envelope and apex-targeting neutralizing antibody lineage provides benchmarks for vaccine design. Cell Rep 23:3249–3261. 10.1016/j.celrep.2018.05.046. 29898396 PMC6019700

[58] Krebs SJ, Kwon YD, Schramm CA, Law WH, Donofrio G, Zhou KH, Gift S, Dussupt V, Georgiev IS, Schätzle S, McDaniel JR, Lai Y-T, Sastry M, Zhang B, Jarosinski MC, Ransier A, Chenine AL, Asokan M, Bailer RT, Bose M, Cagigi A, Cale EM, Chuang G-Y, Darko S, Driscoll JI, Druz A, Gorman J, Laboune F, Louder MK, McKee K, Mendez L, Moody MA, O'Sullivan AM, Owen C, Peng D, Rawi R, Sanders-Buell E, Shen C-H, Shiakolas AR, Stephens T, Tsybovsky Y, Tucker C, Verardi R, Wang K, Zhou J, Zhou T, Georgiou G, Alam SM, Haynes BF, Rolland M, et al. 2019. Longitudinal analysis reveals early development of three MPER-directed neutralizing antibody lineages from an HIV-1-infected individual. Immunity 50:677–691.e13. 10.1016/j.immuni.2019.02.008. 30876875 PMC6555550

[59] Wang C, Schlub TE, Yu WH, Tan CS, Stefic K, Gianella S, Smith DM, Lauffenburger DA, Chaillon A, Julg B. 2022. Landscape of HIV neutralization susceptibilities across tissue reservoirs. Clin Infect Dis 75:1342–1350. 10.1093/cid/ciac164. 35234862 PMC9555844

[60] Zolla-Pazner S, Cardozo T. 2010. Structure–function relationships of HIV-1 envelope sequence-variable regions refocus vaccine design. Nat Rev Immunol 10:527–535. 10.1038/nri2801. 20577269 PMC3167078

[61] Munro JB, Gorman J, Ma X, Zhou Z, Arthos J, Burton DR, Koff WC, Courter JR, Smith AB, Kwong PD, Blanchard SC, Mothes W. 2014. Conformational dynamics of single HIV-1 envelope trimers on the surface of native virions. Science 346:759–763. 10.1126/science.1254426. 25298114 PMC4304640

[62] Silver ZA, Dickinson GM, Seaman MS, Desrosiers RC. 2019. A highly unusual V1 region of env in an elite controller of HIV infection. J Virol 93. 10.1128/JVI.00094-19. PMC6498048 30842322

[63] Gristick HB, Wang H, Bjorkman PJ. 2017. X-ray and EM structures of a natively glycosylated HIV-1 envelope trimer. Acta Crystallogr D Struct Biol 73:822–828. 10.1107/S2059798317013353. 28994411 PMC5633907

[64] Stanfield RL, Berndsen ZT, Huang R, Sok D, Warner G, Torres JL, Burton DR, Ward AB, Wilson IA, Smider VV. 2020. Structural basis of broad HIV neutralization by a vaccine-induced cow antibody. Sci Adv 6:eaba0468. 10.1126/sciadv.aba0468. 32518821 PMC7253169

[65] Yuan M, Cottrell CA, Ozorowski G, van Gils MJ, Kumar S, Wu NC, Sarkar A, Torres JL, de Val N, Copps J, Moore JP, Sanders RW, Ward AB, Wilson IA. 2019. Conformational plasticity in the HIV-1 fusion peptide facilitates recognition by broadly neutralizing antibodies. Cell Host Microbe 25:873–883.e5. 10.1016/j.chom.2019.04.011. 31194940 PMC6579543

[66] Chan K-W, Luo CC, Lu H, Wu X, Kong X-P. 2021. A site of vulnerability at V3 crown defined by HIV-1 bNAb M4008_N1. Nat Commun 12:1–12. 10.1038/s41467-021-26846-z. 34753944 PMC8578649

[67] Barnes CO, Gristick HB, Freund NT, Escolano A, Lyubimov AY, Hartweger H, West AP, Cohen AE, Nussenzweig MC, Bjorkman PJ. 2018. Structural characterization of a highly-potent V3-glycan broadly neutralizing antibody bound to natively-glycosylated HIV-1 envelope. Nat Commun 9:1–12. 10.1038/s41467-018-03632-y. 29593217 PMC5871869

[68] Ripoll DR, Chaudhury S, Wallqvist A. 2021. Using the antibody-antigen binding interface to train image-based deep neural networks for antibody-epitope classification. PLoS Comput Biol 17:e1008864. 10.1371/journal.pcbi.1008864. 33780441 PMC8032195

[69] Cheng H. 2018. Understanding antigen and innate immune recognition: decoding natural antibody diversity with machine learning. Dartmouth College. Hanover, NH.

[70] Miho E, Yermanos A, Weber CR, Berger CT, Reddy ST, Greiff V. 2018. Computational strategies for dissecting the high-dimensional complexity of adaptive immune repertoires. Front Immunol 9:224. 10.3389/fimmu.2018.00224. 29515569 PMC5826328

[71] Schorcht A, Cottrell CA, Pugach P, Ringe RP, Han AX, Allen JD, van den Kerkhof TLGM, Seabright GE, Schermer EE, Ketas TJ, Burger JA, van Schooten J, LaBranche CC, Ozorowski G, de Val N, Bader DLV, Schuitemaker H, Russell CA, Montefiori DC, van Gils MJ, Crispin M, Klasse PJ, Ward AB, Moore JP, Sanders RW. 2022. The glycan hole area of HIV-1 envelope trimers contributes prominently to the induction of autologous neutralization. J Virol 96:e0155221. 10.1128/JVI.01552-21. 34669426 PMC8754230

[72] Ringe RP, Pugach P, Cottrell CA, LaBranche CC, Seabright GE, Ketas TJ, Ozorowski G, Kumar S, Schorcht A, van Gils MJ, Crispin M, Montefiori DC, Wilson IA, Ward AB, Sanders RW, Klasse PJ, Moore JP. 2019. Closing and opening holes in the glycan shield of HIV-1 envelope glycoprotein SOSIP trimers can redirect the neutralizing antibody response to the newly unmasked epitopes. J Virol 93. 10.1128/JVI.01656-18. PMC6363999 30487280

[73] Caskey M, Klein F, Lorenzi JCC, Seaman MS, West AP, Buckley N, Kremer G, Nogueira L, Braunschweig M, Scheid JF, Horwitz JA, Shimeliovich I, Ben-Avraham S, Witmer-Pack M, Platten M, Lehmann C, Burke LA, Hawthorne T, Gorelick RJ, Walker BD, Keler T, Gulick RM, Fätkenheuer G, Schlesinger SJ, Nussenzweig MC. 2015. Viraemia suppressed in HIV-1-infected humans by broadly neutralizing antibody 3BNC117. Nature 522:487–491. 10.1038/nature14411. 25855300 PMC4890714

[74] Gaebler C, Nogueira L, Stoffel E, Oliveira TY, Breton G, Millard KG, Turroja M, Butler A, Ramos V, Seaman MS, Reeves JD, Petroupoulos CJ, Shimeliovich I, Gazumyan A, Jiang CS, Jilg N, Scheid JF, Gandhi R, Walker BD, Sneller MC, Fauci A, Chun T-W, Caskey M, Nussenzweig MC. 2022. Prolonged viral suppression with anti-HIV-1 antibody therapy. Nature 606:368–374. 10.1038/s41586-022-04597-1. 35418681 PMC9177424

[75] Sneller MC, Blazkova J, Justement JS, Shi V, Kennedy BD, Gittens K, Tolstenko J, McCormack G, Whitehead EJ, Schneck RF, Proschan MA, Benko E, Kovacs C, Oguz C, Seaman MS, Caskey M, Nussenzweig MC, Fauci AS, Moir S, Chun T-W. 2022. Combination anti-HIV antibodies provide sustained virological suppression. Nature 606:375–381. 10.1038/s41586-022-04797-9. 35650437 PMC11059968

[76] Ndung’u T, Dong KL, Kwon DS, Walker BD. 2018. A FRESH approach: combining basic science and social good. Sci Immunol 3:eaau2798. 10.1126/sciimmunol.aau2798. 30217812 PMC7593829

[77] Dong KL, Moodley A, Kwon DS, Ghebremichael MS, Dong M, Ismail N, Ndhlovu ZM, Mabuka JM, Muema DM, Pretorius K, Lin N, Walker BD, Ndung'u T. 2018. Detection and treatment of Fiebig stage I HIV-1 infection in young at-risk women in South Africa: a prospective cohort study. Lancet HIV 5:e35–e44. 10.1016/S2352-3018(17)30146-7. 28978417 PMC6506720

[78] Rodrigo AG, Goracke PC, Rowhanian K, Mullins JI. 1997. Quantitation of target molecules from polymerase chain reaction-based limiting dilution assays. AIDS Res Hum Retroviruses 13:737–742. 10.1089/aid.1997.13.737. 9171217

[79] Salazar-Gonzalez JF, Bailes E, Pham KT, Salazar MG, Guffey MB, Keele BF, Derdeyn CA, Farmer P, Hunter E, Allen S, Manigart O, Mulenga J, Anderson JA, Swanstrom R, Haynes BF, Athreya GS, Korber BTM, Sharp PM, Shaw GM, Hahn BH. 2008. Deciphering human immunodeficiency virus type 1 transmission and early envelope diversification by single-genome amplification and sequencing. J Virol 82:3952–3970. 10.1128/JVI.02660-07. 18256145 PMC2293010

[80] Gibson DG, Young L, Chuang R-Y, Venter JC, Hutchison CA, Smith HO. 2009. Enzymatic assembly of DNA molecules up to several hundred kilobases. Nat Methods 6:343–345. 10.1038/nmeth.1318. 19363495

[81] Wei Q, Hargett AA, Knoppova B, Duverger A, Rawi R, Shen C-H, Farney SK, Hall S, Brown R, Keele BF, Heath SL, Saag MS, Kutsch O, Chuang G-Y, Kwong PD, Moldoveanu Z, Raska M, Renfrow MB, Novak J. 2020. Glycan positioning impacts HIV-1 Env glycan-shield density, function, and recognition by antibodies. Iscience 23:101711. 10.1016/j.isci.2020.101711. 33205023 PMC7649354

[82] Kumar S, Stecher G, Li M, Knyaz C, Tamura K. 2018. MEGA X: molecular evolutionary genetics analysis across computing platforms. Mol Biol Evol 35:1547–1549. 10.1093/molbev/msy096. 29722887 PMC5967553

[83] Kumar S, Tamura K, Nei M. 2004. MEGA3: integrated software for molecular evolutionary genetics analysis and sequence alignment. Brief Bioinform 5:150–163. 10.1093/bib/5.2.150. 15260895

[84] Montefiori D. 2018. Protocol for neutralizing antibody screening assay for HIV-1 in TZM-bl cells.

[85] Montefiori DC. 2004. Evaluating neutralizing antibodies against HIV, SIV, and SHIV in luciferase reporter gene assays. Current Protocols in Immunology 64:12.11.1–12.11.17. 10.1002/0471142735.im1211s64. 18432938

[86] Lee JS, Cole SR, Achenbach CJ, Dittmer DP, Richardson DB, Miller WC, Mathews C, Althoff KN, Moore RD, Eron JJ, Center for AIDS Research (CFAR) Network of Integrated Clinical Systems (CNICS). 2018. Cancer risk in HIV patients with incomplete viral suppression after initiation of antiretroviral therapy. PLoS One 13:e0197665. 10.1371/journal.pone.0197665. 29870537 PMC5988275

[87] Dahl V, Gisslen M, Hagberg L, Peterson J, Shao W, Spudich S, Price RW, Palmer S. 2014. An example of genetically distinct HIV type 1 variants in cerebrospinal fluid and plasma during suppressive therapy. J Infect Dis 209:1618–1622. 10.1093/infdis/jit805. 24338353 PMC3997583

[88] Bagaya BS, Vega JF, Tian M, Nickel GC, Li Y, Krebs KC, Arts EJ, Gao Y. 2015. Functional bottlenecks for generation of HIV-1 intersubtype Env recombinants. Retrovirology 12:1–17. 10.1186/s12977-015-0170-8. 25997955 PMC4445978

[89] Dingens AS, Arenz D, Weight H, Overbaugh J, Bloom JD. 2019. An antigenic atlas of HIV-1 escape from broadly neutralizing antibodies distinguishes functional and structural epitopes. Immunity 50:520–532.e3. 10.1016/j.immuni.2018.12.017. 30709739 PMC6435357

