# Supplementary material for: Subtle Longitudinal Alterations in Env Sequence Potentiate Differences in Sensitivity to Broadly Neutralizing Antibodies following Acute HIV-1 Subtype C Infection
Source: J Virol. 2022 Dec 1;96(24):e01270-22. doi: 10.1128/jvi.01270-22 (PMC9769376; doi:10.1128/jvi.01270-22)
Supplement: Supplemental file 1 — Fig. S1 to S4. Download jvi.01270-22-s0001.pdf, PDF file, 1.4 MB [file jvi.01270-22-s0001.pdf]

Subtle longitudinal alterations in Env sequence potentiate differences in sensitivity to broadly neutralizing antibodies following acute HIV-1 subtype C infection

Tawanda Mandizvo[a,b], Nombali Gumede[b], Bongiwe Ndlovu[b], Siphiwe Ndlovu[b], Jaclyn K. Mann[b], Denis Chopera[a,b], Lanish Singh[a,b], Krista L. Dong[c], Bruce D. Walker[b,c], Zaza M. Ndhlovu[a,b,c], Christy L. Lavine[d], Michael S. Seaman[d], Kamini Gounder[a,b] and Thumbi Ndung'u[a,b,c,e*]

[a]Africa Health Research Institute, Durban, South Africa; [b]HIV Pathogenesis Programme, The Doris Duke Medical Research Institute, University of KwaZulu-Natal, Durban, South Africa; [c]Ragon Institute of Massachusetts General Hospital, Massachusetts Institute of Technology and Harvard University, Boston, MA, United States of America; [d]Center for Virology and Vaccine Research, Beth Israel Deaconess Medical Center, Boston, MA, United States of America; [e]Division of Infection and Immunity, University College London, London, United Kingdom.

*Corresponding Author
Africa Health Research Institute, 719 Umbilo Road, Durban, 4001, South Africa
Phone: +27 31 260 4887; Email: thumbi.ndungu@ahri.org

SUPPLEMENTARY MATERIAL

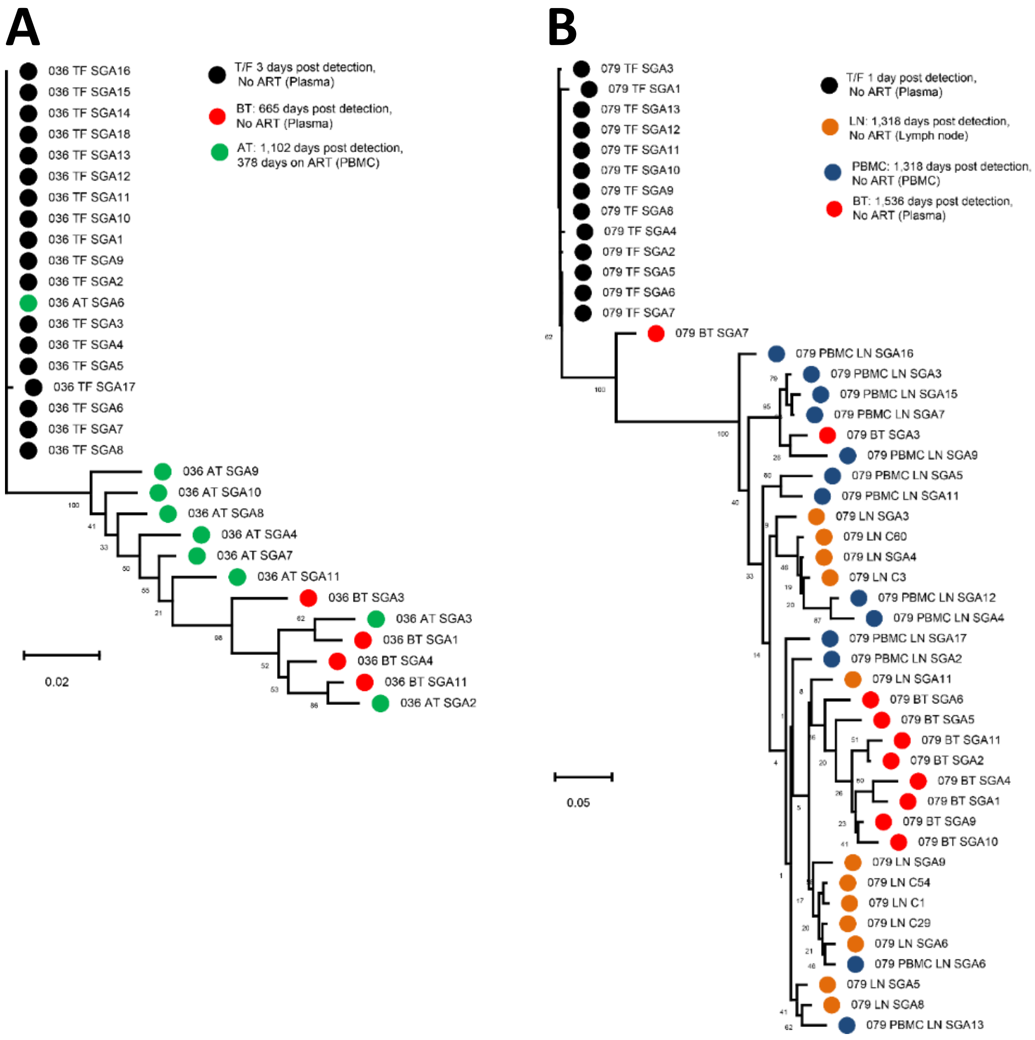

A
036 TF SGA16
036 TF SGA15
036 TF SGA14
036 TF SGA18
036 TF SGA13
036 TF SGA12
036 TF SGA11
036 TF SGA10
036 TF SGA1
036 TF SGA9
036 TF SGA2
036 AT SGA6
036 TF SGA3
036 TF SGA4
036 TF SGA5
036 TF SGA17
036 TF SGA6
036 TF SGA7
036 TF SGA8
036 AT SGA9
036 AT SGA10
036 AT SGA8
036 AT SGA4
036 AT SGA7
036 AT SGA11
036 BT SGA3
036 AT SGA3
036 BT SGA1
036 BT SGA4
036 BT SGA11
036 AT SGA2
T/F 3 days post detection, No ART (Plasma)
BT: 665 days post detection, No ART (Plasma)
AT: 1,102 days post detection, 378 days on ART (PBMC)
0.02
B
079 TF SGA3
079 TF SGA1
079 TF SGA13
079 TF SGA12
079 TF SGA11
079 TF SGA10
079 TF SGA9
079 TF SGA8
079 TF SGA4
079 TF SGA2
079 TF SGA5
079 TF SGA6
079 TF SGA7
079 BT SGA7
079 PBMC LN SGA16
079 PBMC LN SGA3
079 PBMC LN SGA15
079 PBMC LN SGA7
079 BT SGA3
079 PBMC LN SGA9
079 PBMC LN SGA5
079 PBMC LN SGA11
079 LN SGA3
079 LN C60
079 LN SGA4
079 LN C3
079 PBMC LN SGA12
079 PBMC LN SGA4
079 PBMC LN SGA17
079 PBMC LN SGA2
079 LN SGA11
079 BT SGA6
079 BT SGA5
079 BT SGA11
079 BT SGA2
079 BT SGA4
079 BT SGA1
079 BT SGA9
079 BT SGA10
079 LN SGA9
079 LN C54
079 LN C1
079 LN C29
079 LN SGA6
079 PBMC LN SGA6
079 LN SGA5
079 LN SGA8
079 PBMC LN SGA13
T/F 1 day post detection, No ART (Plasma)
LN: 1,318 days post detection, No ART (Lymph node)
PBMC: 1,318 days post detection, No ART (PBMC)
BT: 1,536 days post detection, No ART (Plasma)
0.05

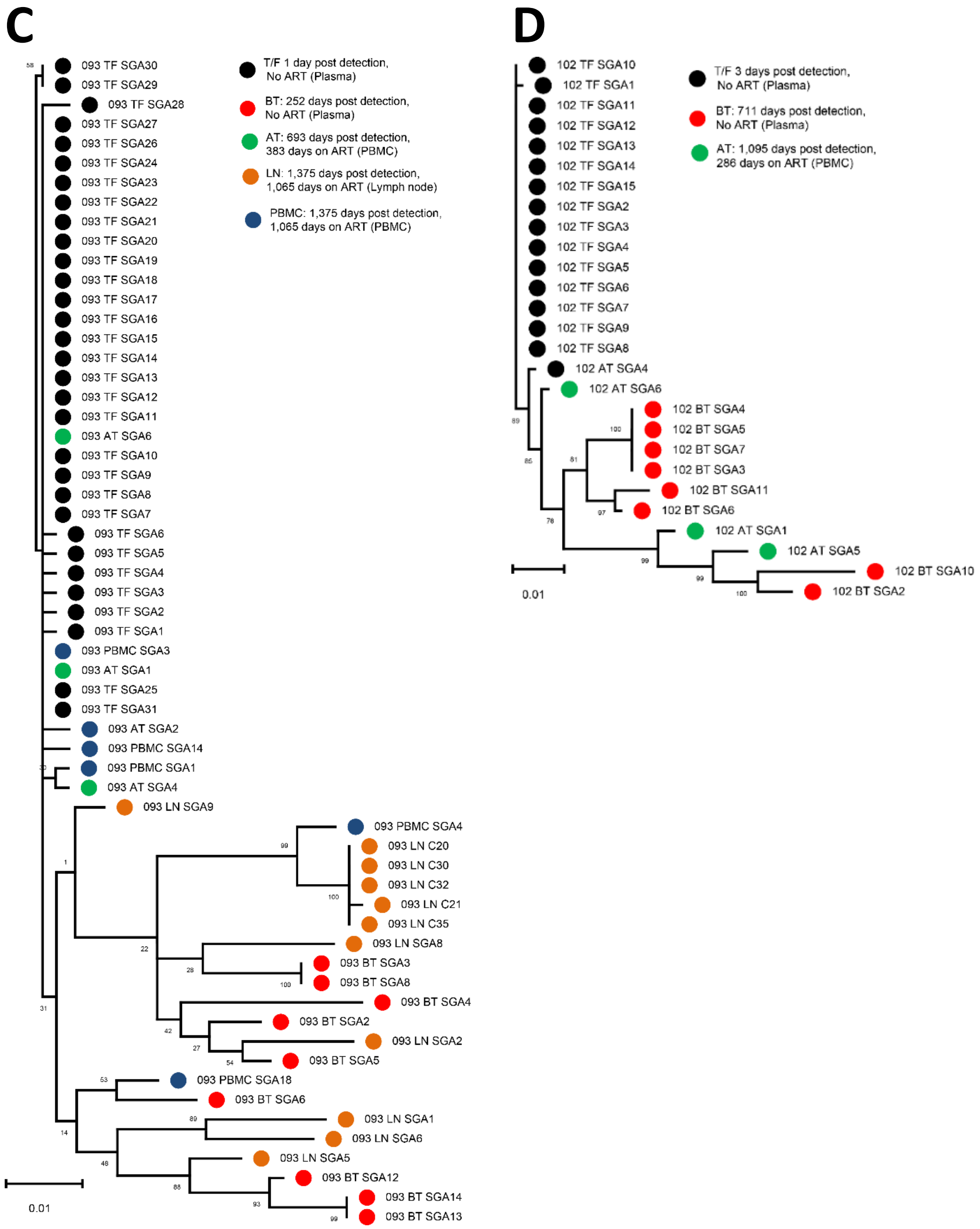
C
093 TF SGA30
093 TF SGA29
093 TF SGA28
093 TF SGA27
093 TF SGA26
093 TF SGA24
093 TF SGA23
093 TF SGA22
093 TF SGA21
093 TF SGA20
093 TF SGA19
093 TF SGA18
093 TF SGA17
093 TF SGA16
093 TF SGA15
093 TF SGA14
093 TF SGA13
093 TF SGA12
093 TF SGA11
093 AT SGA6
093 TF SGA10
093 TF SGA9
093 TF SGA8
093 TF SGA7
093 TF SGA6
093 TF SGA5
093 TF SGA4
093 TF SGA3
093 TF SGA2
093 TF SGA1
093 PBMC SGA3
093 AT SGA1
093 TF SGA25
093 TF SGA31
093 AT SGA2
093 PBMC SGA14
093 PBMC SGA1
093 AT SGA4
093 LN SGA9
093 PBMC SGA4
093 LN C20
093 LN C30
093 LN C32
093 LN C21
093 LN C35
093 LN SGA8
093 BT SGA3
093 BT SGA8
093 BT SGA4
093 BT SGA2
093 LN SGA2
093 BT SGA5
093 PBMC SGA18
093 BT SGA6
093 LN SGA1
093 LN SGA6
093 LN SGA5
093 BT SGA12
093 BT SGA14
093 BT SGA13
0.01
T/F 1 day post detection, No ART (Plasma)
BT: 252 days post detection, No ART (Plasma)
AT: 693 days post detection, 383 days on ART (PBMC)
LN: 1,375 days post detection, 1,065 days on ART (Lymph node)
PBMC: 1,375 days post detection, 1,065 days on ART (PBMC)
D
102 TF SGA10
102 TF SGA1
102 TF SGA11
102 TF SGA12
102 TF SGA13
102 TF SGA14
102 TF SGA15
102 TF SGA2
102 TF SGA3
102 TF SGA4
102 TF SGA5
102 TF SGA6
102 TF SGA7
102 TF SGA9
102 TF SGA8
102 AT SGA4
102 AT SGA6
102 BT SGA4
102 BT SGA5
102 BT SGA7
102 BT SGA3
102 BT SGA11
102 BT SGA6
102 AT SGA1
102 AT SGA5
102 BT SGA10
102 BT SGA2
0.01
T/F 3 days post detection, No ART (Plasma)
BT: 711 days post detection, No ART (Plasma)
AT: 1,095 days post detection, 286 days on ART (PBMC)

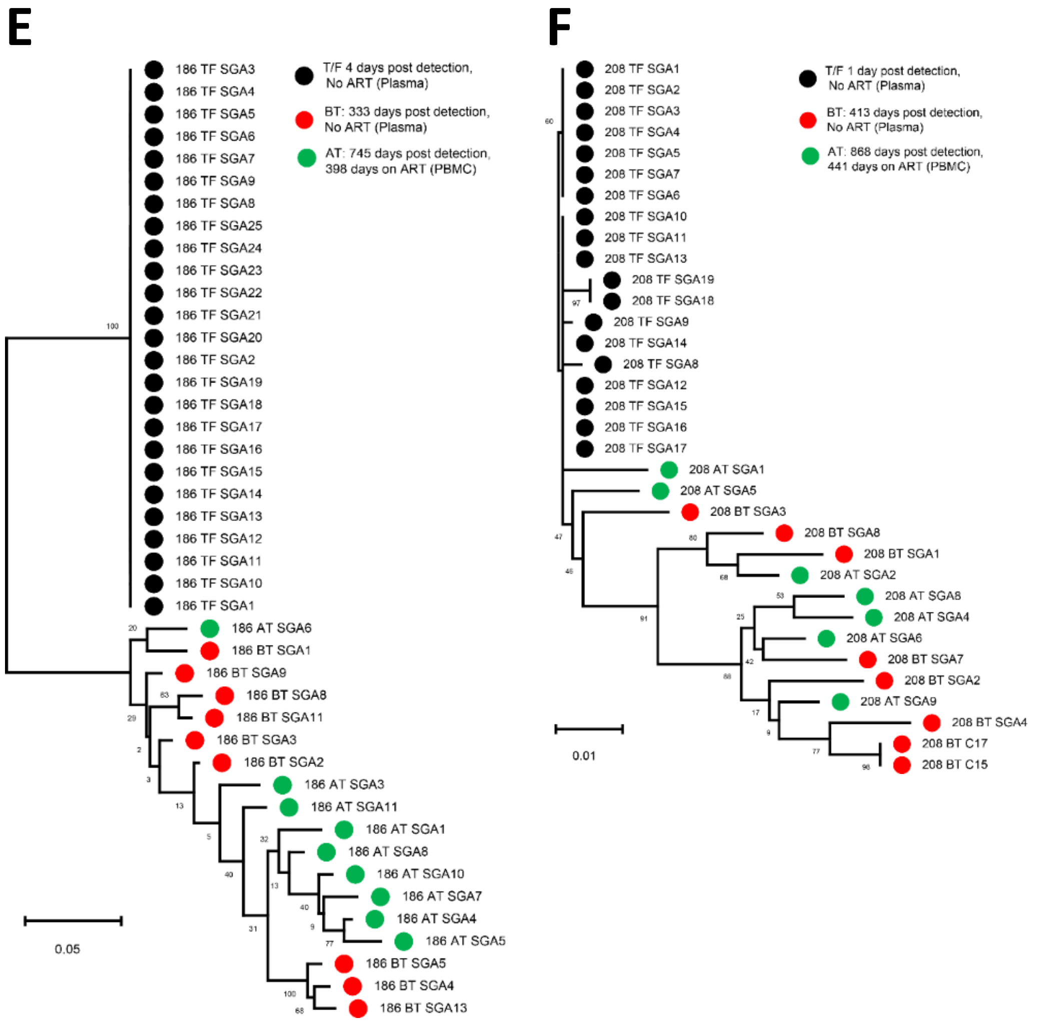
E
186 TF SGA3
186 TF SGA4
186 TF SGA5
186 TF SGA6
186 TF SGA7
186 TF SGA9
186 TF SGA8
186 TF SGA25
186 TF SGA24
186 TF SGA23
186 TF SGA22
186 TF SGA21
186 TF SGA20
186 TF SGA2
186 TF SGA19
186 TF SGA18
186 TF SGA17
186 TF SGA16
186 TF SGA15
186 TF SGA14
186 TF SGA13
186 TF SGA12
186 TF SGA11
186 TF SGA10
186 TF SGA1
186 AT SGA6
186 BT SGA1
186 BT SGA9
186 BT SGA8
186 BT SGA11
186 BT SGA3
186 BT SGA2
186 AT SGA3
186 AT SGA11
186 AT SGA1
186 AT SGA8
186 AT SGA10
186 AT SGA7
186 AT SGA4
186 AT SGA5
186 BT SGA5
186 BT SGA4
186 BT SGA13
T/F 4 days post detection, No ART (Plasma)
BT: 333 days post detection, No ART (Plasma)
AT: 745 days post detection, 398 days on ART (PBMC)
0.05
F
208 TF SGA1
208 TF SGA2
208 TF SGA3
208 TF SGA4
208 TF SGA5
208 TF SGA7
208 TF SGA6
208 TF SGA10
208 TF SGA11
208 TF SGA13
208 TF SGA19
208 TF SGA18
208 TF SGA9
208 TF SGA14
208 TF SGA8
208 TF SGA12
208 TF SGA15
208 TF SGA16
208 TF SGA17
208 AT SGA1
208 AT SGA5
208 BT SGA3
208 BT SGA8
208 BT SGA1
208 AT SGA2
208 AT SGA8
208 AT SGA4
208 AT SGA6
208 BT SGA7
208 BT SGA2
208 AT SGA9
208 BT SGA4
208 BT C17
208 BT C15
T/F 1 day post detection, No ART (Plasma)
BT: 413 days post detection, No ART (Plasma)
AT: 868 days post detection, 441 days on ART (PBMC)
0.01

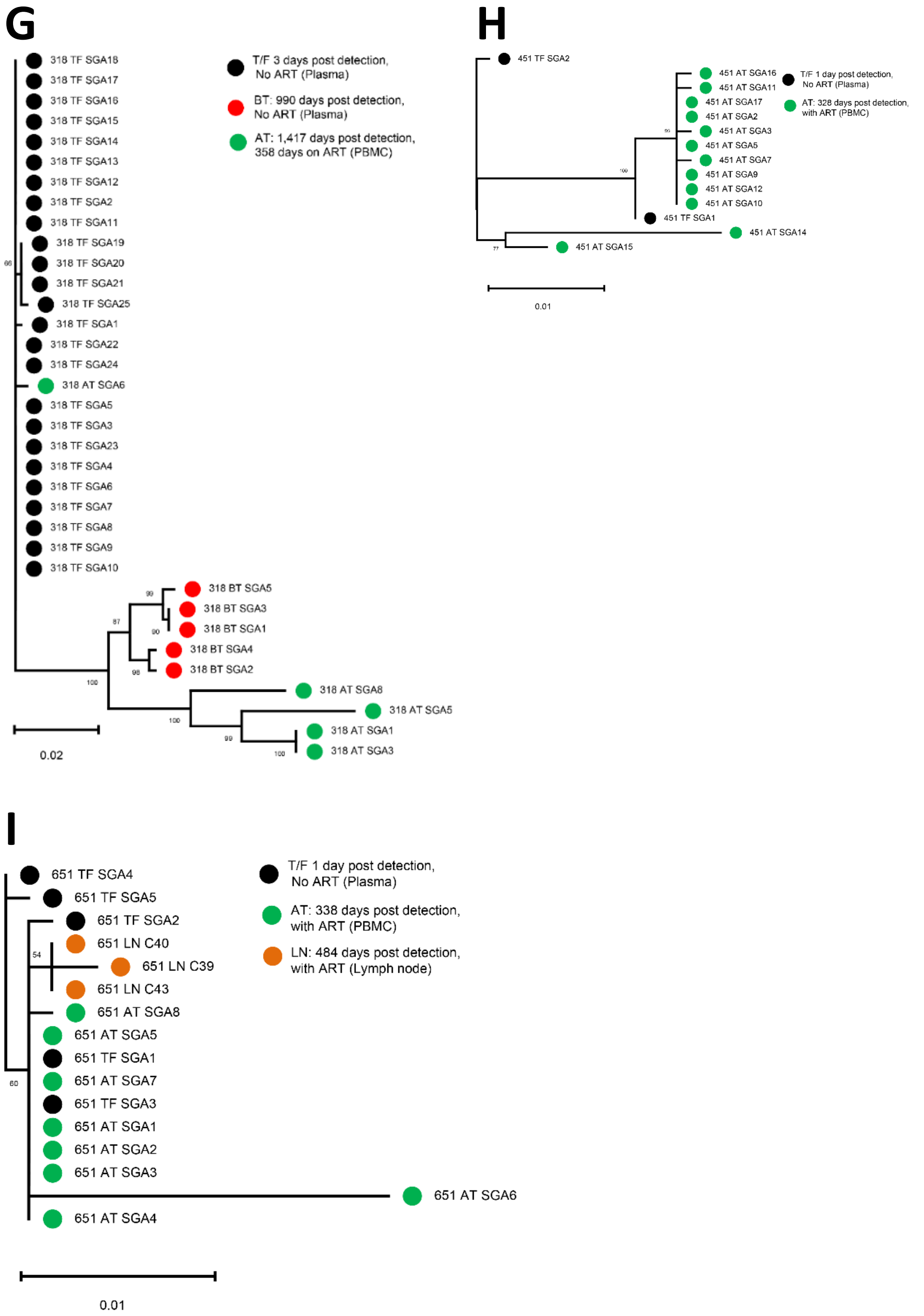

Supplementary Figure S1. Maximum likelihood phylogenetic trees of full-length subtype C HIV Env amino acid sequences from the studied nine participants. Transmitter/founder sequences are shown with black labels, chronic sequences (before ART) are denoted by red labels, while PBMC reservoir sequences (~1year after ART) are denoted by green labels. In some participants (079, 093 and 651), lymph node derived sequences are denoted by orange labels, whereas their matching timepoint PBMC derived sequences

are denoted by blue labels. The branch length is drawn to scale so that the relatedness between different sequences can be readily assessed, with branch lengths measured in the number of substitutions per site. Model/Method = Jones-Taylor-Thornton (JTT) model; Test of phylogeny = Bootstrap method; No. of Bootstrap replications =100.

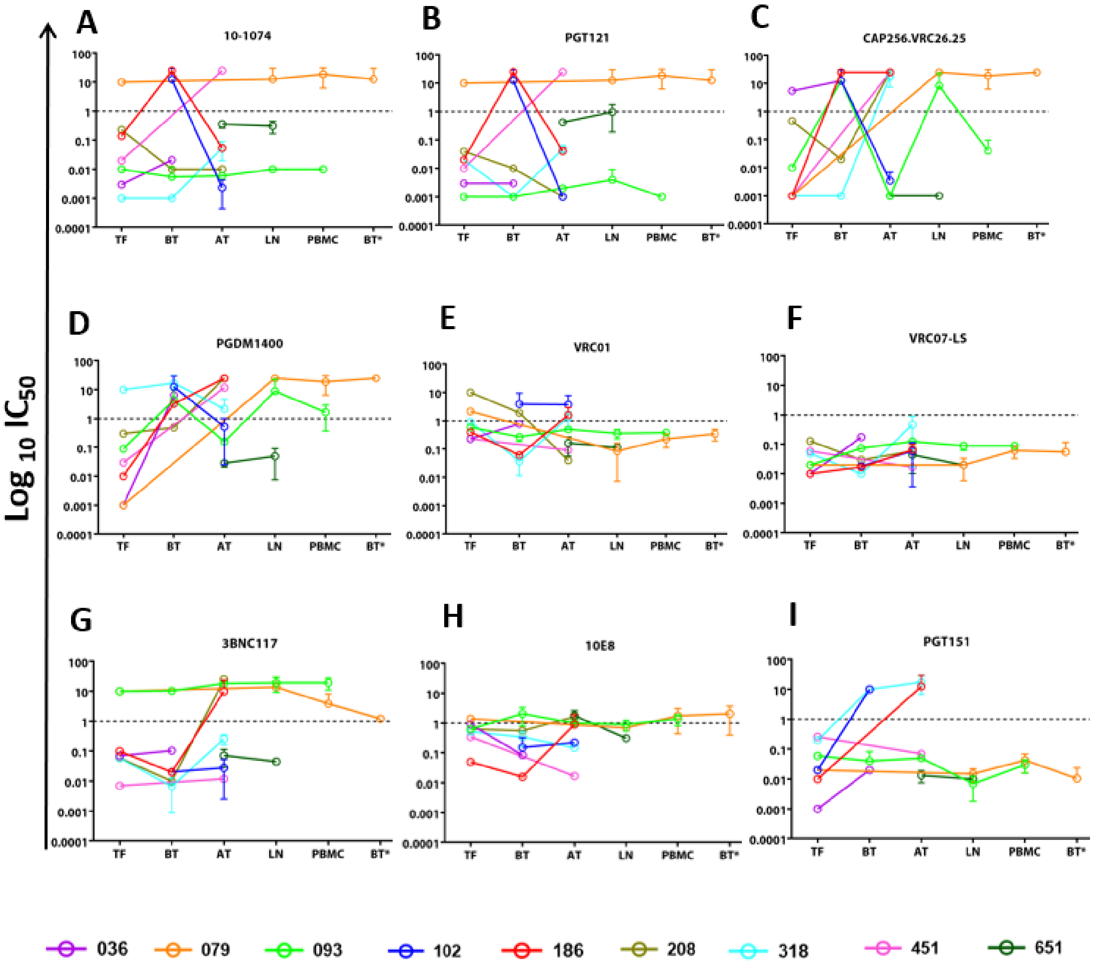

Supplementary Figure S2. Line graphs showing distribution of intra-participant viral neutralization sensitivity against the tested bNAbs over time. A) 10-1074; B) PGT121; C) CAP256.VRC26.25; D) PGDM1400; E) VRC01; F) VRC07.LS; G)3BNC117; H) 10E8; I) PGT151. Each color-coded line represents a different participant. The mean neutralization titer against each antibody is represented as a circle for each time point. Y-axis: $\text{Log}_{10}\,\text{IC}_{50}$; X-axis: sampled compartment and the relative days post observed plasma viremia increase from left to right (TF = transmitted/founder; BT = before treatment; AT = ~1 year after treatment initiation; LN = lymph node; and PBMC = sequences from PMBC at timepoint matching lymph node excision). *Denotes sequences obtained from participant 079 only who remained ART naïve throughout the study.

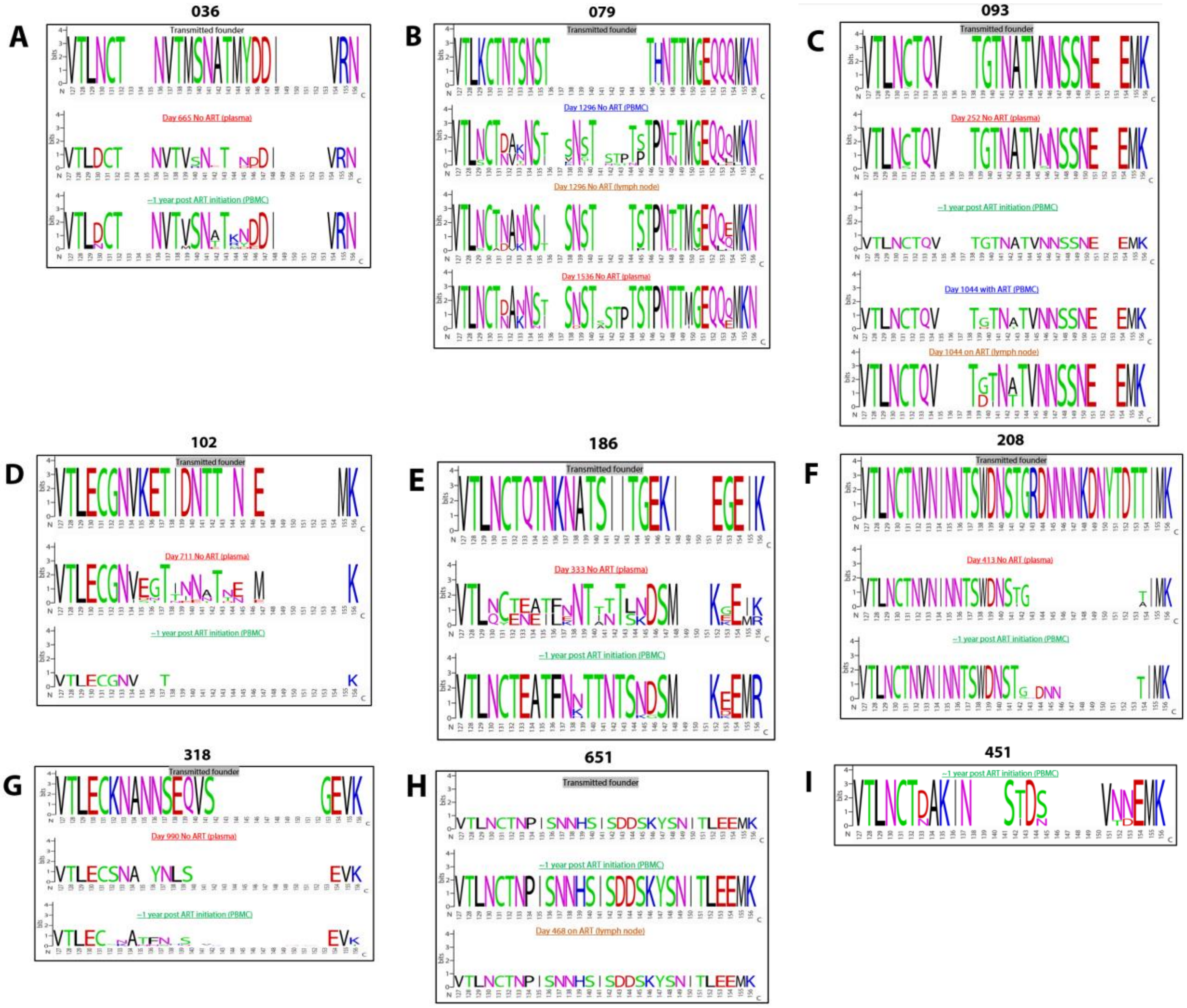

Supplementary Figure S3. Logo plots showing longitudinal intraparticipant V1 sequence variation. Two PIDs (451 & 651) are acutely treated. Seven PIDs (079, 093, 186, 036, 102, 208 & 318) are chronically treated. Each panel figure represents sequences derived from the same participant, with the top-most logo plot sequence representing the earliest sampling time point and the bottom-most representing the latest sampled timepoint. The amino acid colors do not denote anything, they are just for presentation purposes. The sequence numbering is based on the HXB2 numbering. The height of each amino acid residue highlights the enrichments and depletions at the particular sequence position per sampled timepoint.

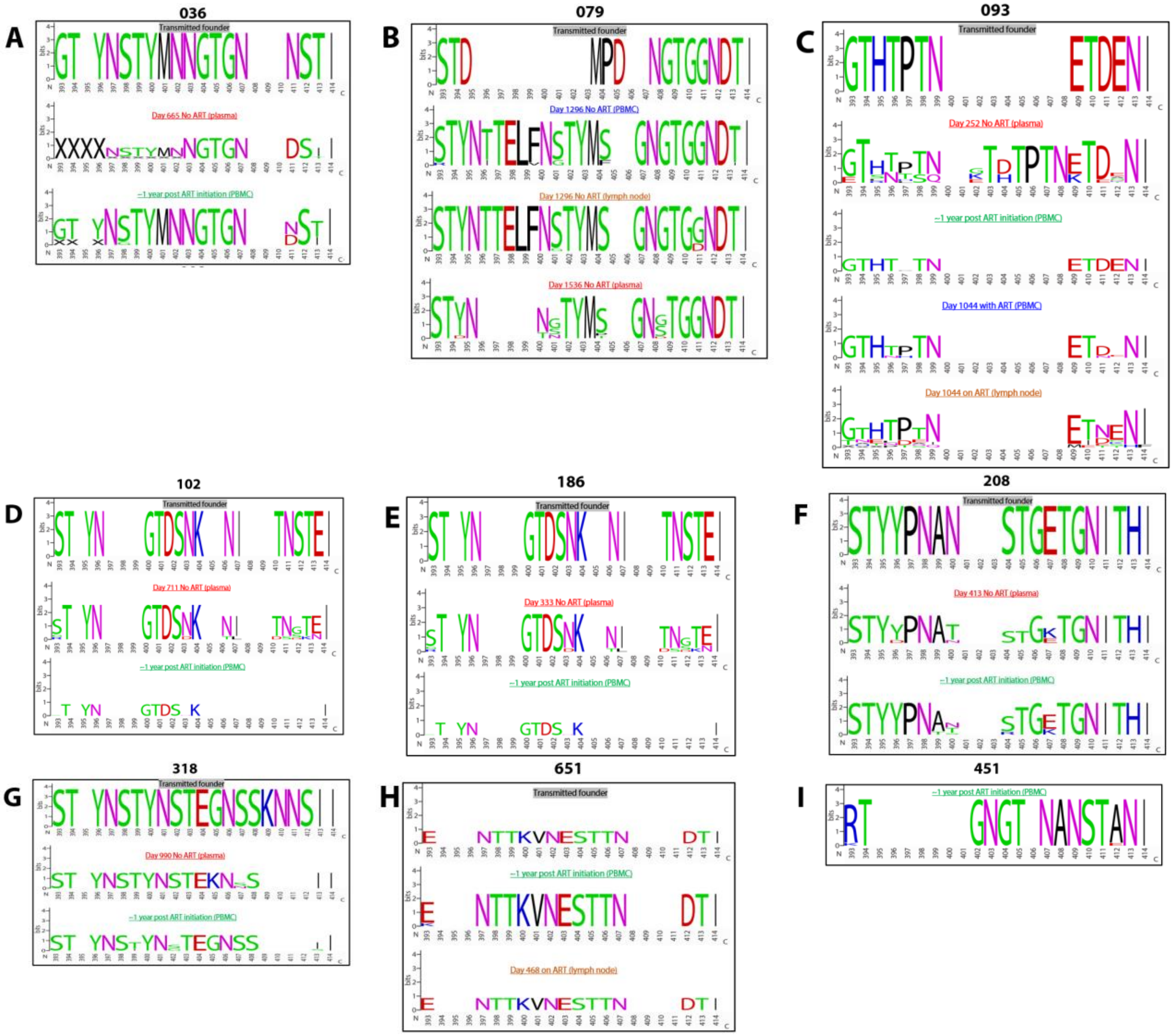

Supplementary Figure S4. Logo plots showing longitudinal intraparticipant V4 sequence variation. Two PIDs (451 & 651) are acutely treated. Seven PIDs (079, 093, 186, 036, 102, 208 & 318) are chronically treated. Each panel figure represents sequences derived from the same participant, with the top-most logo plot sequence representing the earliest sampling time point and the bottom-most representing the latest sampled timepoint. The amino acid colors do not denote anything, they are just for presentation purposes. The sequence numbering is based on the HXB2 numbering. The height of each amino acid residue highlights the enrichments and depletions at the particular sequence position per sampled timepoint.
